# Myelodysplastic Syndrome associated *TET2* mutations affect NK cell function and genome methylation

**DOI:** 10.1038/s41467-023-36193-w

**Published:** 2023-02-03

**Authors:** Maxime Boy, Valeria Bisio, Lin-Pierre Zhao, Fabien Guidez, Bérénice Schell, Emilie Lereclus, Guylaine Henry, Juliette Villemonteix, Fernando Rodrigues-Lima, Katia Gagne, Christelle Retiere, Lise Larcher, Rathana Kim, Emmanuelle Clappier, Marie Sebert, Arsène Mekinian, Olivier Fain, Anne Caignard, Marion Espeli, Karl Balabanian, Antoine Toubert, Pierre Fenaux, Lionel Ades, Nicolas Dulphy

**Affiliations:** 1 Université Paris Cité, Institut de Recherche Saint Louis, EMiLy, INSERM UMR_S1160, F-75010 Paris, France; 2 Institut Carnot OPALE, Institut de Recherche Saint-Louis, Hôpital Saint-Louis, F-75010 Paris, France; 3 CNRS, GDR3697 “Microenvironment of tumor niches”, Micronit, F-75010 Paris, France; 4 Université Paris Cité, Institut de Recherche Saint Louis INSERM UMR_S1131, F-75010 Paris, France; 5 Laboratoire d’Immunologie et d’Histocompatibilité, Assistance Publique des Hôpitaux de Paris (AP-HP), Hôpital Saint-Louis, F-75010 Paris, France; 6 Universite Paris Cité, Sorbonne Paris Cite, Unité BFA, CNRS UMR 8251, 75013 Paris, France; 7 Etablissement Français du Sang, Centre Pays de la Loire, F-44011 Nantes, France; 8 Université de Nantes, INSERM UMR1307, CNRS UMR 6075, CRCI2NA team 12, F-44000 Nantes, France; 9 LabEx IGO « Immunotherapy, Graft, Oncology », F-44000 Nantes, France; 10 LabEx Transplantex, Université de Strasbourg, 67000 Strasbourg, France; 11 Laboratoire d’Hématologie, Hôpital Saint-Louis, AP-HP, F-75010 Paris, France; 12 Department d’Hématologie Sénior, Hôpital Saint-Louis, AP-HP, F-75010 Paris, France; 13 Université Paris Cité, Institut de Recherche Saint Louis INSERM UMR_944, F-75010 Paris, France; 14 Service de Medecine Interne, Hôpital Saint-Antoine, AP-HP, F-75012 Paris, France; 15 Departement Hospitalo-Universitaire Inflammation-Immunopathologie-Biotherapie, Sorbonne Université, Hôpital de la Pitié-Salpêtrière, F-75013 Paris, France

**Keywords:** NK cells, Myelodysplastic syndrome, Translational immunology, Immunosurveillance

## Abstract

Myelodysplastic syndromes (MDS) are clonal hematopoietic disorders, representing high risk of progression to acute myeloid leukaemia, and frequently associated to somatic mutations, notably in the epigenetic regulator *TET2*. Natural Killer (NK) cells play a role in the anti-leukemic immune response via their cytolytic activity. Here we show that patients with MDS clones harbouring mutations in the *TET2* gene are characterised by phenotypic defects in their circulating NK cells. Remarkably, NK cells and MDS clones from the same patient share the *TET2* genotype, and the NK cells are characterised by increased methylation of genomic DNA and reduced expression of Killer Immunoglobulin-like receptors (KIR), perforin, and TNF-α. In vitro inhibition of TET2 in NK cells of healthy donors reduces their cytotoxicity, supporting its critical role in NK cell function. Conversely, NK cells from patients treated with azacytidine (#NCT02985190; https://clinicaltrials.gov/) show increased KIR and cytolytic protein expression, and IFN-γ production. Altogether, our findings show that, in addition to their oncogenic consequences in the myeloid cell subsets, *TET2* mutations contribute to repressing NK-cell function in MDS patients.

## Abstract (web summary)

Myelodysplastic syndromes are characterised by clonal haematopoiesis, with the affected cells often harbouring mutations in the *TET2* gene, an important regulator of DNA methylation state. Here authors show that the same mutations are also found in NK cells, perturbing their DNA methylation pattern and cytolytic function.

## Introduction

Myelodysplastic Syndromes (MDS) are clonal myeloid malignancies characterized by ineffective hematopoiesis and bone marrow (BM) failure leading to blood cytopenias and a high risk of transformation to Acute Myeloid Leukemia (AML)^1^. MDS patients with a high risk of AML progression (“high-risk MDS” according to the international prognostic scoring system) are treated with hypomethylating agents (HMA) including azacitidine (AZA) and decitabine (DAC) which can increase overall survival and reduce AML progression^2,3^. Mutations in *TET2* and *IDH1/2* genes, involved in the epigenetic regulation of gene transcription, are common genetic alterations found in MDS^1^. Whereas *TET2* encodes for a methylcytosine dioxygenase catalyzing the oxidation of the methyl group of 5-methycytosine (5mC) to 5-hydroxymethylcytosine (5-hmC), the isocitrate dehydrogenases (IDH) 1 and 2 control the production of α-ketoglutarate (αKG), a metabolite required for TET2 activity^4^. Most *TET2* mutations described are loss of function and are generally mutually exclusive with *IDH1/2* mutations. By contrast, *IDH1/2* mutations in MDS lead to a new enzymatic activity that generates 2-hydroxyglutarate (2HG) in place of αKG and inhibits TET2 activity^5^. Therefore, *TET2* and *IDH1/2* mutations lead to hypermethylation of the genomic DNA in myeloid cells^6^. MDS patients carrying mutations in *TET2* are more likely to respond to HMA treatment^7^, but mechanisms of action of HMA on MDS cells are still unclear and could involve the BM microenvironment, including immune (lymphocytes, myeloid-derived suppressive cells)^8^ and non-immune partners (mesenchymal stromal cells)^9^.

Natural Killer (NK) lymphocytes are key players in the anti-leukemic response. NK cell targets malignant cells by direct cytotoxicity through the release of perforin, granzymes, engagement of death receptors, or secreting cytokines like IFN-γ and TNF-α^10,11^. Their function is regulated by a balance between inhibitory (KIR, NKG2A) and activating (NKp30, NKp46, DNAM-1, NKG2D) receptors. We and others have shown defects in NK cell phenotype and function in AML and MDS patients^12–15^, but mechanisms leading to deficient NK cells are still unclear. Moreover, whether the enhanced sensitivity to HMA treatment of MDS patients with *TET2/IDH*^*MUT*^ relies on the restoration of NK-cell activity is unknown.

In this study, we show MDS *TET2/IDH* mutations are carried by NK cells and associated with defects in phenotype and function. Those perturbations rely on genome-wide hypermethylation of DNA, leading to the reduced expression of key molecules for NK cell function. Finally, HMA treatment normalizes the expression of NK cell-related genes in vitro as well as in vivo. Altogether, our results established that TET2 regulates NK-cell biology, and suggest that HMA treatments in *TET2/IDH* mutated patients could also be beneficial by restoring the functionality of NK cells.

## Results

### MDS/CMML patients with *TET2/IDH* mutations display a distinct NK cell phenotype

Blood NK cells from 33 MDS/CMML patients (myelodysplastic syndromes and chronic myelomonocytic leukemia, respectively, Supplementary Data 1) and 23 HD were phenotyped by flow cytometry. Surface expression of maturation markers (CD69, CD57, KLRG1), activating (NKp30, NKp46, DNAM-1, NKG2D) and inhibitory (CD96, KIR2D, CD85j) receptors was investigated (Supplementary Table 1). Small perturbations were observed on circulating NK cells of MDS/CMML individuals compared with HD in the expression of some activating (DNAM-1, NKp46) and inhibitory (CD96) receptors. Surprisingly, an almost 2-fold reduction of the killer immunoglobulin-like receptors (KIR) expression was found in MDS patients’ NK cells compared to HD (31.2% and 54.8%, respectively, *p* < 0.01, Supplementary Table 1).

**Fig. 1:**
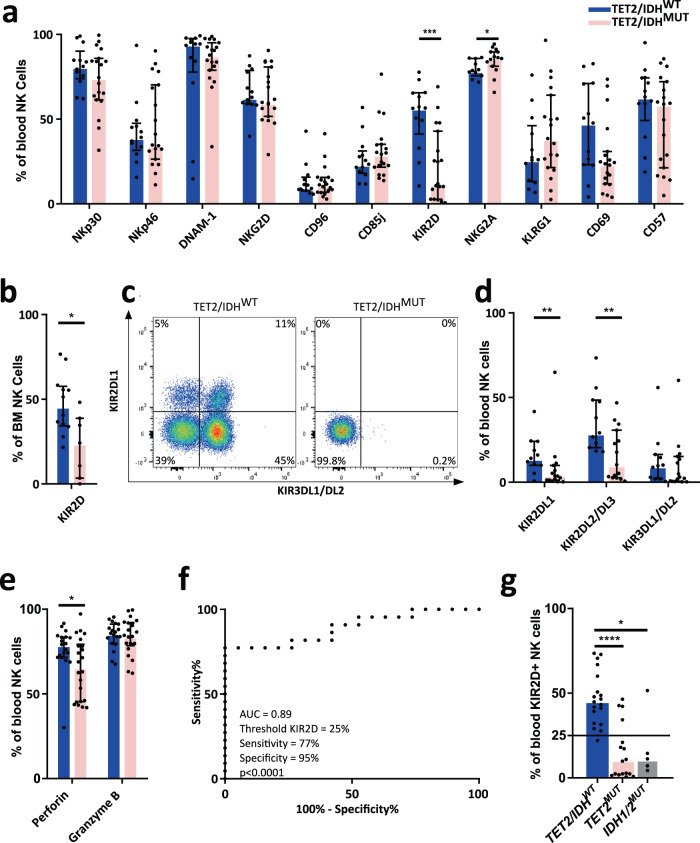
*TET2/IDH* mutations in MDS/CMML patients lead to the reduction of KIR and Perforin expression in NK cells. **a** Percentages of blood NK cells expressing activating (NKp30, NKp46, DNAM-1, NKG2D) and inhibitory (CD96, CD85j, KIR2D, NKG2A) receptors, and maturation/activation markers (KLRG1, CD69, CD57) were measured by flow cytometry in *TET2/IDH*^WT^ (*n* = 13, *n* = 11 for NKG2A) and *TET2/IDH*^MUT^ (*n* = 19, *n* = 15 for NKG2A) MDS/CMML patients. Statistics were calculated with the nonparametric Mann–Whitney test, two-sided, KIR2D ***p = 0.0005, NKG2A *p = 0.0246. **b** Percentages of KIR2D^+^ BM NK cells measured by flow cytometry in *TET2/IDH*^WT^ (*n* = 12) and *TET2/IDH*^MUT^ (*n* = 7) MDS/CMML patients. Statistics were calculated with the nonparametric Mann–Whitney test two-sided *p = 0.013. **c** and **d** Specific expression of KIR2DL1, KIR2DL2/DL3, and KIR3DL1/DL2 in blood NK cells detected by flow cytometry in *TET2/IDH*^WT^ (*n* = 11) and *TET2/IDH*^MUT^ (*n* = 15) MDS/CMML patients. One representative example is shown in **c**. Statistics were calculated with the nonparametric Mann–Whitney test, KIR2DL1 **p = 0.0092, two-sided KIR2DL2/DL3 **p = 0.0077. **e** Intracellular perforin and granzyme B expression in blood NK cells measured by flow cytometry in *TET2/IDH*^WT^ (*n* = 19) and *TET2/IDH*^MUT^ (*n* = 22) MDS/CMML patients. Statistics were calculated with the nonparametric Mann–Whitney test *p = 0.0337. **f** Receiver operating characteristic (ROC) curve depicting the relationship of true *TET2* mutation presence (sensitivity) and false *TET2* mutation presence (100%-specificity) for a KIR2D expression threshold at 25% in blood NK cells (*p* < 0.0001) quantified by flow cytometry in *TET2/IDH*^WT^ (*n* = 19) and *TET2*^MUT^ (*n* = 17) MDS/CMML patients. **g** KIR2D expression on blood NK cells of the *TET2/IDH*^*WT*^ (*n* = 19), *TET2*^*MUT*^ (*n* = 17) and *IDH*^*MUT*^ (*n* = 5) patients showed in the ROC curve. The horizontal black bar indicates the threshold at 25% KIR2D^+^ NK cells. Statistics were calculated with the nonparametric Mann–Whitney test, *TET2/IDH*^*WT*^ vs. *TET2*^*MUT*^: ****p < 0.0001; *TET2/IDH*^*WT*^ vs. *IDH*^*MUT*^: *p = 0.0152. For all the analysis, data are presented as medians and interquartile ranges. Source data are provided as a Source Data file.

We asked whether patients with mutations affecting DNA methylation (i.e., mutations in *TET2*, *IDH1*, or *IDH2*) could exhibit specific NK-cell phenotypes (Fig. 1, Supplementary Table 1). Interestingly, a deep loss in the expression of KIRs with 2 Ig-like extracellular domains (KIR2D) was observed in patients with *TET2/IDH* mutations compared to *TET2/IDH*^*WT*^ on blood (*n* = 19 vs. 13, median 11.9% vs. 56.5%, *p* < 0.001, Fig. 1a) and BM NK cells (*n* = 7 vs. 12, median 24.2% vs. 45.9% *p* < 0.05, Fig. 1b). In parallel, the inhibitory receptor NKG2A was significantly increased on peripheral NK cells in *TET2/IDH*^*MUT*^ patients compared to *TET2/IDH*^*WT*^ (*n* = 15 vs. 11, 86.3% vs. 78.7%, *p* = 0.025, Fig. 1A, Supplementary Table 2), underlying the balance between KIRs and NKG2A expression^16^. Of note, the KIR-positive T-cell subset was not affected by the *TET2/IDH* mutational status in patients (Supplementary Fig. 1). Importantly, censuring the few patients treated with either drug to stimulate the erythro-thrombopoiesis (EPO *n* = 4 out of 63 patients, TPO *n* = 1) or immunomodulating molecules (lenalidomide *n* = 4, glucocorticoid *n* = 4) did not modify our observations demonstrating that treatments were not at the origin of the decrease in KIR expression (Supplementary Fig. 2).

KIRs are encoded in a 15 genes locus with high variability in gene content across individuals^17^. To investigate if *TET2/IDH* mutations could differentially affect *KIR* genes expression, the KIR locus was genotyped for 22 MDS/CMML patients, and KIR surface expression was analyzed for genes highly present with KIR2DL1, KIR2DL2/DL3, and KIR3DL1/DL2 specific mAbs (Fig. 1c, d, Supplementary Fig. 3 and Table 3). We confirmed a specific reduction of KIR2DL1 (3.6% vs. 14.1%, *p* < 0.01) and KIR2DL2/DL3 (10.3% vs. 29.1%, *p* < 0.01) on NK cells of *TET2/IDH*^*MUT*^ patients compared to WT (*n* = 15 vs. *n* = 11, respectively) (Fig. 1c, d, Supplementary Table 2). By contrast, KIR3DL1/DL2 expression was not significantly modified by the *TET2/IDH* mutation status (Fig. 1d), despite the strong reduction observed in some patients (Fig. 1c, d).

We next quantified perforin and granzyme B expression in NK cells of MDS/CMML patients as surrogate markers of NK-cell cytolytic capacity. We observed a reduced percentage of perforin-positive NK cells in *TET2/IDH*^*MUT*^ patients (*p* < 0.05), whereas granzyme B expression was not affected (Fig. 1e). This loss of intracellular perforin was correlated with reduced KIR (KIR2D and KIR3DL1/DS1) expression at the NK-cell surface in *TET2/IDH*^*MUT*^ patients (*r* = 0.65, *p* = 0.001; Supplementary Fig. 4). Indeed, the loss of perforin expression was mainly observed in KIR^−^ NK cells whereas KIR^+^ NK-cells were not affected (Supplementary Fig. 5), suggesting a co-regulation of KIR and perforin expressions.

Then, we reasoned that the TET2 function may be the main parameter modifying the NK cell phenotype. Therefore, we analyzed the performance of KIR2D expression as a classifier of patients with or without *TET2* mutations by calculating a receiver operation characteristic (ROC) curve using KIR2D expression on circulating NK cells in *TET2/IDH*^*WT*^ (*n* = 19) and *TET2*^*MUT*^ (*n* = 17) patients. A maximum area under the curve (AUC) equal to 0.89 predicted that a threshold of 25% of KIR2D^+^ NK cells allowed classifying mutated and not-mutated patients with a specificity of 95% and a sensitivity of 77% (*p* < 0.0001, Fig. 1f). Studying KIR2D expression separately in *TET2*^*MUT*^ and *IDH1*/2^MUT^ patients, we observed that 4 out of 5 patients mutated for either *IDH1* or *IDH2* showed a comparable decrease of KIR2D expression than *TET2*^*MUT*^ individuals (Fig. 1g). This observation confirmed that *IDH1/2* mutations could phenocopy *TET2* mutations at least when considering NK cell phenotype.

Altogether, our results unraveled a strong relationship between the presence of mutations in *TET2* and *IDH1/2* genes in MDS/CMML patients and the alterations in NK-cell phenotype. To gain further insights into the molecular mechanisms accounting for this loss of NK cell function, we focused our study on *TET2* mutated patients, excluding the *IDH* mutated patients from our cohort, and interrogated whether NK and MDS cells could share *TET2* mutations.

### *TET2* mutations observed in MDS/CMML patients at diagnosis can be found in blood NK-cells

All MDS/CMML patients recruited in this study were analyzed at diagnosis of the disease for the presence of somatic mutations in 80 genes usually found mutated in myeloid malignancies (Supplementary Table 4). As expected, *TET2*^*MUT*^ and *TET2/IDH*^*WT*^ patients could show mutations in additional genes frequently observed in MDS/CMML^1^, including *SRSF2* (29% and 3%, respectively), *SF3B1* (18% and 20%, respectively) or *ASXL1* (18% and 17%, respectively) (Supplementary Fig. 6 and Supplementary Table 5). Of note, *TET2*^*MUT*^ patients presented a mean of 2 mutations in *TET2* (range 1–4 mutations).

**Fig. 2:**
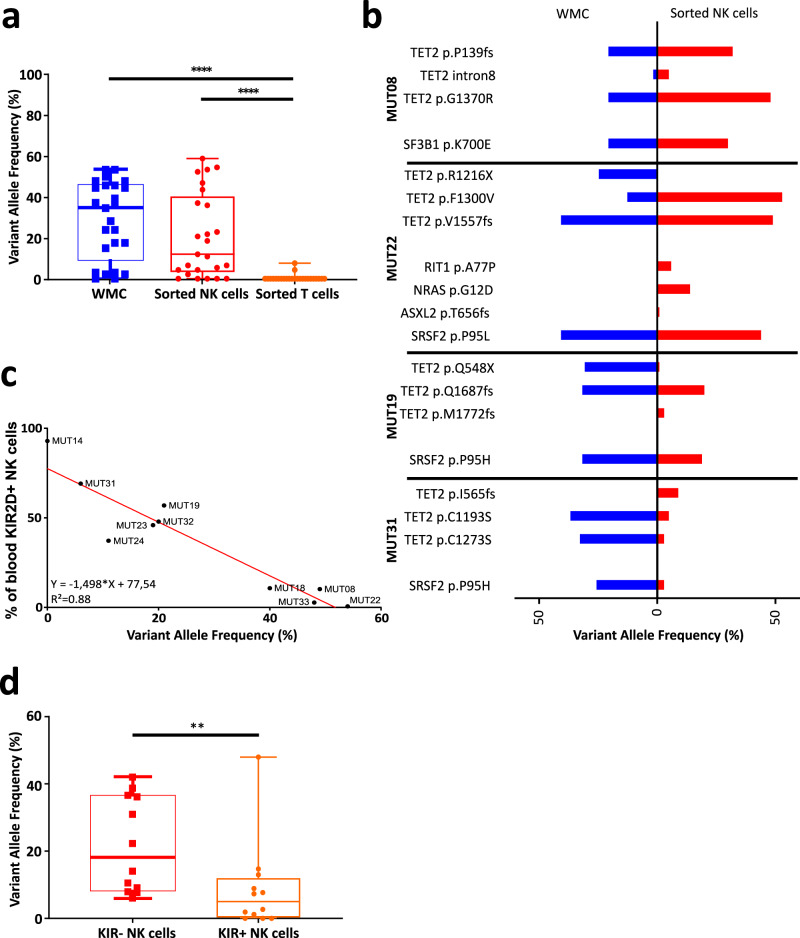
*TET2* mutations identified in bulk cells of MDS patients at diagnosis are also observed in NK cells and correlate with KIR2D expression. **a** The percentage of Variant Allele Frequency (VAF) for the mutations of *TET2* observed in the white mononuclear cells (WMC) and in sorted NK/T cells at diagnosis of *TET2*^MUT^ MDS/CMML patients (*n* = 10) was evaluated by NGS analysis (with a range from 1 to 4 mutations per patient). Data are represented as box-and-whisker plots (minimum VAF, 25% percentile, median, 75% percentile, and maximum VAF respectively for WMC: 0%, 11.25%, 32.5%, 42.5%, 49%; for NK cells: 0%, 3.5%, 14%, 35.5%, 54%; and for T cells: 0%, 0%, 0%, 0%, 7%). Nonparametric two-sided Wilcoxon matched-pairs signed rank test was used to determine statistical significance. ****p < 0.0001. **b** VAF (%) of the different mutations detected in the WMC at diagnosis (blue) and in sorted NK cells (red) in 4 patients (MUT08, MUT22, MUT19, MUT31; see Supplementary Table 6 for more information). **c** Correlation curve between the percentage of KIR2D^+^ NK cells and the *TET2* VAF (%) in blood NK cells from the MDS/CMML patients analyzed in (**a**). Linear regression was calculated, *r* = 0.88, *p* < 0.0001. **d** The VAF percentage in the WMC and in sorted KIR+ and KIR− NK cells at diagnosis from *TET2*^MUT^ MDS/CMML patients (*n* = 5, range from 1 to 4 mutations per patient) was evaluated by NGS analysis. Data are represented as box-and-whisker plots (minimum VAF, 25% percentile, median, 75% percentile, and maximum VAF respectively for KIR− NK cells: 6.1%, 8.3%, 18.2%, 36.5%, 42.1%; and KIR+ NK cells: 0%, 1.2%, 7.3%, 13%, 48%). Statistics were calculated with a two-sided nonparametric Wilcoxon matched-pairs signed rank test. *p = 0.0186. Source data are provided as a Source Data file.

Thus, we analyzed whether MDS-associated mutations were present in NK cells. PBMC of 10 MDS patients diagnosed with mutations in *TET2* among other genes were sorted for NK cells. Nine out of 10 patients showed some mutations in NK cells shared with the blood or BM white mononuclear cell (WMC) samples at diagnosis, including in *TET2* and other genes (Supplementary Table 6). The variant allele frequencies (VAF) for *TET2* mutations were not significantly different in NK cells (median = 11%, range 0–54%) compared to WMC (median = 32%, range 2–49%, Fig. 2a). By contrast, consistent with their unchanged phenotype previously observed, MDS-associated mutations were rarely found in T cells (Supplementary Table 6, Supplementary Fig. 7A), and with a VAF below 7% when present (Fig. 2a). Importantly, whereas 7 patients presented a strict concordance in the mutation profiles between analyses on WMC and NK cells (Fig. 2b and Supplementary Table 6), 3 patients (MUT19, MUT22, MUT31) showed mutations in NK cells unobserved in WMC (Fig. 2b).

Furthermore, we observed a strong correlation between the expression of KIR2D receptor and the VAF of *TET2* mutations in NK cells (Fig. 2c, *R*^2^ = 0.88, *p* < 0.0001). In the 10 patients analyzed by NGS, those with a percentage of KIR2D^+^ NK-cells below 25% showed a *TET2* mutation VAF higher than 20% in the WMC at diagnosis and higher or equal to 34% in the NK-cell subset. To confirm this observation, we selected 5 patients with sufficient amounts of KIR2D^+^ and KIR2D^−^ NK cells and performed an NGS analysis on cells sorted according to the expression of the KIR2D and KIR3DL1/DL2 (hereafter designed has KIR^+^ or KIR^−^ NK cells). Importantly, 4 out of 5 patients showed a high percentage of KIR2D^+^ cells, superior to the threshold of 25% we have calculated previously (Fig. 1F). As expected, patients showed higher *TET2*^*MUT*^ VAF in the KIR^−^ (median = 18.2%, range 6.1–42.1%) than the KIR^+^ cells (median = 5%, range 0–48%, *p* < 0.01, Fig. 2d, Supplementary Fig. 7B). In addition, KIR^−^ NK cell subset showed *TET2* mutations leading to non-functional TET2 proteins in all patients tested. In detail, patients MUT08, MUT19, and MUT23 presented frameshift mutations, leading to premature stop-codon, with VAFs ranging from 22% to 37% in KIR^−^ cells, as compared to a range from 2% to 15% in the KIR^+^ cells. Patients MUT24 and MUT32 showed missense mutations in the TET2 catalytic domain, with VAFs ranging from 11% to 39% in the KIR^−^ cells as compared to 1% to 7% in the KIR^+^ cells. Moreover, two patients (MUT19 and MUT23) showed a shared nonsense mutation at position 548 only present in the KIR^−^ NK cells. Mutations in other genes were also enriched in the KIR^−^ population (Supplementary Table 6). Altogether, these observations suggested that *TET2* mutations can be found in NK cells and are related to reduced KIR expression in *TET2*^*MUT*^ MDS/CMML patients.

### TET2 directly binds to and regulates KIR locus expression in NK cells

**Fig. 3:**
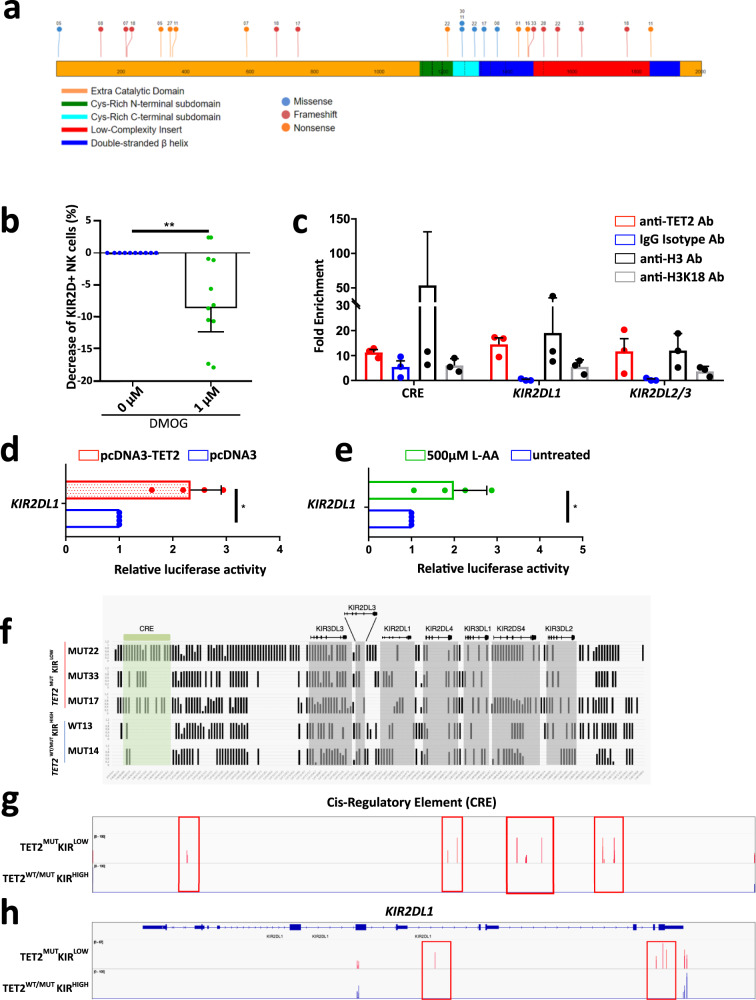
TET2 regulates KIR expression on NK cells through its binding onto and regulating methylation of the KIR locus. **a** *TET2* mutational landscape in the 13 *TET2*^*MUT*^ *patients* with KIR2D expression below 25% established with the St Jude protein paint software (https://proteinpaint.stjude.org/). Mutations were classified as Missense (blue), Frameshift (red), and Nonsense (orange). Numbers associated with each mutation designed patients (Supplementary Data 1). **b** Percentage of KIR2D^+^ NK cells after 5 days of in vitro treatment with 1 µM DMOG (*n* = 10). DMSO alone was used as a control. One dot represents one PBMC sample. Nonparametric two-sided Wilcoxon matched-pairs signed rank test was used to determine statistical significance. **p = 0.0059. Data are presented as median and interquartile range. **c** Fold enrichment of sequences specific for the Cis-regulatory element (CRE), *KIR2DL1* promoter, and *KIR2DL2/3* promoter analyzed in sorted NK cells by ChIP-qPCR with TET2, H3 or H3K18 specific mAbs or IgG isotype control. Means ± SD is shown (*n* = 3). Each dot represents one independent experiment. **d** Luciferase activity in HEK293T cells co-transfected with a *TET2* full-length plasmid or an empty plasmid as control, and the luciferase-reporter construct containing the region (−147 + 60) of the *KIR2DL1* promoter. Means ± SD is shown (*n* = 4). Each dot represents one independent experiment. Nonparametric two-sided Wilcoxon matched-pairs signed rank test was used to determine statistical significance. *p = 0.0286. **e** Jurkat cells transfected with the *KIR2DL1* promoter-luciferase reporter plasmid were treated with 500 µM of L-AA for 16 h and analyzed by detecting luminescence signal. Means ± SD is shown (*n* = 4). Each dot represents one independent experiment. Nonparametric two-sided Wilcoxon matched-pairs signed rank test was used to determine statistical significance. *p = 0.0286. **f** RRBS DNA methylation profiles of the extended KIR locus of NK cells with low or high KIR2D expression. Each graph represents the DNA methylation profile of sorted NK cells from blood samples of 5 patients; vertical bars represent the percentage of DNA methylation at the CpG position. CRE and *KIR* genes were highlighted in green and gray respectively. **g** and **h** IGV (Integrative Genomics Viewer) view of CpG read signals corresponding to DNA methylation in NK cells, based on the high (*TET2*^WT/MUT^KIR^HIGH^, in blue bars) and low (*TET2*^MUT^KIR^LOW^, in red bars) KIR expression. Genome profiles at the CRE region (**g**) and the *KIR2DL1* gene (**h**) loci showed variation in the DNA methylation pattern between the two groups of patient NK cells. Red peaks/boxes show significant differences in the DNA methylation levels at particular CpG positions of these specific loci. Source data are provided as a Source Data file.

To evaluate the role of TET2 in regulating KIR expression, we first analyzed the type and the positioning of the *TET2* mutations found in MDS/CMML patients with a reduced percentage of KIR^+^ NK cells (below 25%) at diagnosis (Fig. 3a). This analysis revealed that all but one patient showed either nonsense or frameshift mutations leading to a premature stop-codon and the absence of a functional TET2 protein, extending the observation made in KIR^−^ NK cells (Supplementary Table 6). The only exception was the patient MUT30 who presented a missense mutation p.R1261H (c.G3782A substitution). This mutation has already been described in the catalog of somatic mutations in cancer (COSMIC) database^18^ with a functional consequence prediction (Functional Analysis through Hidden Markov Models (FATHMM)) score equal to 0.99^19^, forerunning a highly significant functional impact on the TET2 protein.

Therefore, we asked whether the inhibition of the enzymatic function of TET2 in NK cells could also lead to a significant reduction of the KIR2D protein expression. We treated NK cells from healthy individuals with dimethyloxalyl glycine (DMOG), an α-ketoglutarate (α-KG) analog which inhibits α-KG-dependent enzymes including TET2^20^. In line with our results in *TET2*^*MUT*^ MDS patients, TET2 inhibition in DMOG treatment significantly reduced the percentage of KIR2D^+^ NK cells (*p* < 0.001, Fig. 3b), suggesting a role for TET2 in modulating the KIR expression on NK cells or the KIR2D^+^ subset proliferation.

We then sought to determine whether TET2 had a direct impact on *KIR* gene expression. We performed chromatin immunoprecipitation (ChIP) followed by real-time qPCR analysis on sorted NK cells from control PBMC with a TET2-specific monoclonal antibody (mAb) and evaluated the presence of the proximal promoter regions of *KIR2DL1* and *KIR2DL2/3* genes. We also tested TET2 binding site onto a predicted *cis*-regulatory element (CRE) localized upstream of the KIR locus (chr19:54,688,519–54.702.287)^21^. We found that tested KIR promoters and CRE sequences were enriched in TET2-specific ChIP fragments (Fig. 3c).

To substantiate the role of TET2 in driving KIR expression, we generated a luciferase reporter vector containing 210 (−147; +63) nucleotides of the predicted KIR2DL1 promoter associated with the TET2 ChIP assay experiment. Co-transfection of this reporter with a TET2 overexpression vector into the HEK293T cell line is associated with an increase of luciferase activity increased by 2.3-fold compared to the empty vector (Fig. 3d). To corroborate this data, the TET2 expressing Jurkat lymphoid cell line was transfected with the KIR2DL1-luciferase reporter vector and then treated with ascorbic acid (L-AA), a catalyst of the TET2 hydroxylation activity of 5-methylcytosine in DNA and an already-known enhancer of the *KIR* locus demethylation^22,23^. We observed a significant increase of the luciferase signal by 2-fold in the treated condition compared to the control, demonstrating that L-AA enhanced TET2 binding and activity onto the *KIR* promoter sequence (Fig. 3e).

Based on these observations, we anticipated that NK cells with low KIR expression and carrying *TET2* mutations could show perturbations in the methylation profile of the *KIR* locus. Therefore, we performed reduced representation bisulfite sequencing (RRBS) analysis on NK-cells sorted from *TET2*^*WT*^ and *TET2*^*MUT*^ MDS/CMML patients (*n* = 1 and *n* = 4 respectively, Fig. 3f–h) to assess their DNA methylation pattern. Three *TET2*^*MUT*^ MDS/CMML patients were chosen based on a KIR2D expression on NK cells below 25%, and a *TET2* mutation VAF superior to 40% in the cell bulk at diagnosis (*TET2*^*MUT*^ KIR^LOW^ patients: MUT22, MUT27, MUT33). One *TET2*^*MUT*^ patient was chosen because of a KIR2D expression on NK cells at 90% (*TET2*^*MUT*^ KIR^HIGH^ patient: MUT14) and one *TET2*^*WT*^ patient (*TET2*^*WT*^ KIR^HIGH^ patient: WT13) was used as controls.

The analysis of the CpG islands in the CRE sequence and the *KIR2DL1* gene showed increased methylation in the three *TET2*^*MUT*^ KIR^LOW^ patients compared to the *TET2*^*MUT*^ KIR^HIGH^ and the *TET2*^*WT*^ ones (Fig. 3g, h).

Altogether, these findings demonstrate the targeting of the *KIR* locus by TET2 and strongly suggest that loss of TET2 leads to the hypermethylation of the *KIR* locus in NK cells.

### *TET2* mutations lead to hypermethylation of key genes for cytotoxicity and cytokine release by NK cell

**Fig. 4:**
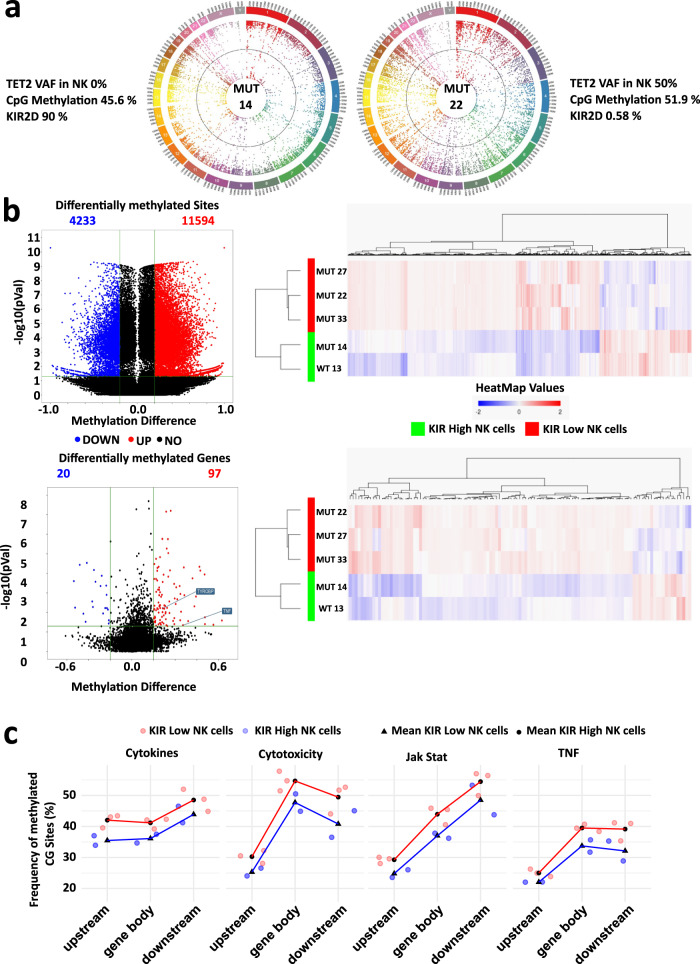
Loss of TET2 leads to DNA hypermethylation and decreases key gene expression for NK cell function. **a** Circos plots showing whole-genome CpG methylation status in patient MUT14 characterized by the absence of *TET2* mutations in NK cells and a high expression of KIR2D, and in patient MUT22 with a VAF of 50% for the *TET2* mutation [NM_001127208:exon11:c,4669_4672del:p.V1557fs] and a very low expression of KIR2D in NK cells. **b** Volcano plots and heatmaps showed the overall increase of global DNA methylation levels reported after RRBS analysis, in the *TET2*^MUT^KIR^LOW^ (*n* = 3) vs. *TET2*^WT/MUT^KIR^HIGH^ (*n* = 2) NK cells. Heatmaps depicted supervised clustering of the significantly modified sited genes between patients’ subgroups. Blue dots/bars show the hypomethylated CpG/genes whereas red dots/bars show the hypermethylated ones (methylation difference ≥20%, unadjusted *p*-value ≤ 0.05). Top panel shows the differentially methylated CpG sites. Bottom panel shows the differentially methylated genes. **c** GO-enrichment analysis on the differentially methylated genes in the *TET2*^MUT^KIR^LOW^ and *TET2*^WT/MUT^KIR^HIGH^ NK cells. Percentages of methylated CpG sites were calculated in gene bodies and 10 kb upstream or downstream of the gene of interest in *TET2*^MUT^KIR^LOW^ and *TET2*^WT/MUT^KIR^HIGH^ NK cells (in red and blue, respectively) and aggregated across all genes of a given KEGG pathway for each sample. Pathways of interest shown are the cytokine–cytokine receptor interactions (KEGG reference hsa04060), the JAK-STAT signaling pathway (hsa04630), the TNF signaling pathway (hsa04668), and the NK cell-mediated cytotoxicity pathway (hsa04650). Source data are provided as a Source Data file.

The analysis of DNA methylation in TET2-deficient mouse models showed widespread DNA hypermethylation, including in enhancer elements^24^. RRBS analyses of NK cells from *TET2*^*MUT*^ KIR^LOW^ MDS/CMML patients confirmed that genomic DNA was more methylated in NK cells with a high VAF of *TET2*^MUT^ and a reduced KIR2D cell surface expression compared to *TET2*^WT^ NK cells (Fig. 4a). We then asked whether *TET2* mutations in NK cells could interfere with the methylation and expression of genes relevant for NK cell function. RRBS analyses of NK cells from *TET2*^*MUT*^ KIR^LOW^ MDS/CMML patients showed significant hypermethylation compared to KIR^HIGH^ patients with 11,594 hypermethylated CpG sites among the 15,827 differentially methylated sites (methylation difference ≥20%, *p* ≤ 0.05) (Fig. 4b, upper panel, Supplementary Data 2). Among the 97 genes found hypermethylated in *TET2*^MUT^KIR^LOW^ patients compared to KIR^HIGH^ patients (Fig. 4b, lower panel, Supplementary Data 3), methylation of *TYROBP* (coding for DAP12) and *TNF* was predicted to significantly inhibit the NK cell-mediated cytotoxicity pathway as defined by the KEGG database (*p* < 0.05, KEGG pathway hsa04650)^25^. Reports suggested variations in methylation by TET2 of the CpG loci according to their position regarding the gene body and the respective regulatory elements^24,26,27^. We analyzed the percentage of 5hmC in the gene bodies and 10 kb upstream or downstream on the whole genome^26^. Then, the mean of methylation has been calculated for genes involved in pathways of interest for NK cell biology. Percentage of 5hmC trends up in *TET2*^MUT^KIR^LOW^ NK cells compared to KIR^HIGH^ samples in coding and flanking regions of genes implicated in the cytokine–cytokine receptor interactions (KEGG pathway reference hsa04060), the JAK-STAT signaling pathway (hsa04630), the TNF signaling pathway (hsa04668) and the NK cell-mediated cytotoxicity (hsa04650) (Fig. 4c), indicating a decrease of these cell functions. Interestingly, the increase of methylation affected the gene bodies as well as their flanking regions, suggesting a differential role for TET2 in NK cells as compared to myeloid cells in which TET2 mainly regulates enhancers^24,27^.

**Fig. 5:**
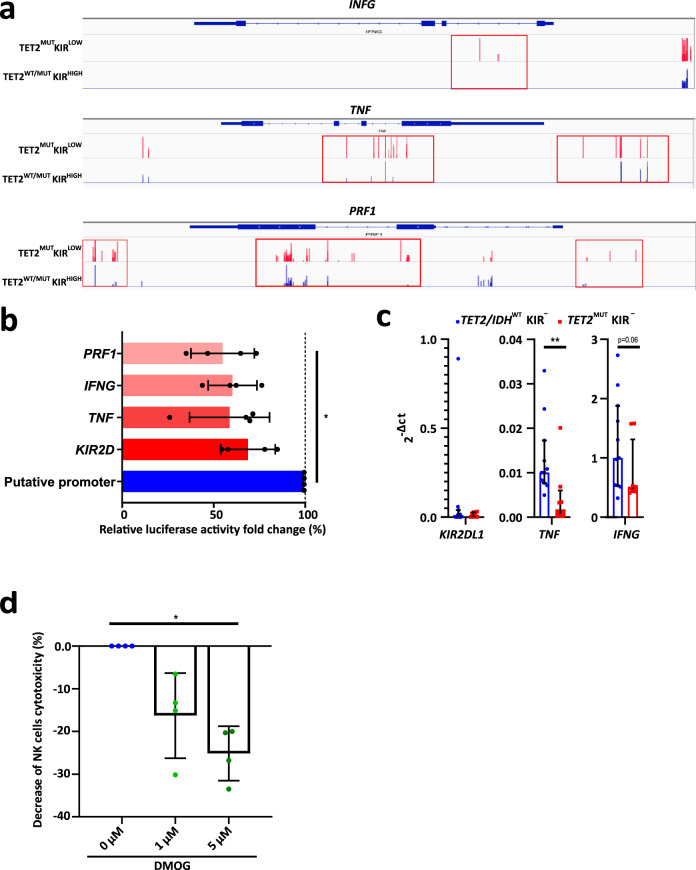
Hypermethylation of particular CpG sites represses NK cell gene expression and function. **a** Methylation profiles, established after RRBS analyses, depicted with IVG at the *IFNG* (upper panel), *TNF* (middle panel) and *PRF1* (lower panel) loci, were shown in NK cells from patients segregated on the high (*TET2*^WT/MUT^KIR^HIGH^, in blue bars) and low (*TET2*^MUT^KIR^LOW^, in red bars) KIR expression. Red peaks/boxes show significant differences in the DNA methylation levels of these specific loci. **b** The regulatory activity of specific CpG sites was analyzed in the HEK293T cell line transfected with a luciferase-reporter plasmid including methylated or non-methylated genes’ regulatory regions. Relative luciferase activities of the in vitro methylated regions were compared to their non-methylated counterpart (FC = Methylated/Putative Promoter). Means ± SD is shown (*n* = 4). Data were analyzed using the one-way Friedman test followed by a Dunn’s test. *p = 0.0433. **c** Quantification of the *KIR2DL1*, *TNF*, and *IFNG* transcripts by RT-qPCR on KIR2D^−^ NK cells sorted from *TET2/IDH*^WT^ (*n* = 11, in blue) and *TET2*^MUT^ (*n* = 10, in red) patients. Medians and interquartile are shown. Statistics were calculated with the two-sided Mann–Whitney test, **p = 0.0036. **d** Fold changes of NK cells killing activity against the NK-sensitive cell line K562 previously treated for 5 days with 1 µM DMOG (*n* = 10). Each dot represents one independent experiment. DMSO alone was used as a control. Means ± SD is shown. Data were analyzed using the one-way Friedman test followed by a Dunn’s test. *p = 0.0417. Source data are provided as a Source Data file.

We then looked at the methylation of *IFNG*, *TNF*, and *PRF1*, three genes determinant in NK cell anti-leukemic activity^28^. RRBS analysis showed hypermethylated CpG islands in the three loci in the *TET2*^*MUT*^KIR^LOW^ patients compared to *TET2*^*MUT*^KIR^HIGH^ or the *TET2*^*WT*^ patients, suggesting a reduced transcription of these genes (Fig. 5a).

To evaluate the functional relevance of DNA methylation in controlling the regulatory regions of these genes, a luciferase reporter assay was performed in the HEK293T cell line (Fig. 5b). Regions of interest, showing differences in their methylation profile between *TET2*^*MUT*^KIR^LOW^ and *TET2*^*MUT*^KIR^HIGH^ NK cells, were cloned into a CpG-free vector upstream of the luciferase gene, without minimal promoter to determine the functional activity of the promoter. The constructs were then methylated in vitro specifically onto the insert sequences. Methylation resulted in significantly decreased luciferase activity compared to the unmodified construct, suggesting that the promoter function of the region of interest is dampened upon DNA methylation (Fig. 5b). In parallel, the decreased expression of *TNF* and *INFG* in presence of *TET2* mutations was confirmed by qRT-PCR in KIR2D^−^ NK cell sorted from *TET2/IDH*^WT^ and *TET2*^MUT^ patients (Fig. 5c).

Altogether, these results showed that key molecules for NK-cell function were under the control of the TET2 demethylation pathway and that their transcription was reduced in *TET2*^MUT^ NK cells. Consequently, we asked whether TET2 inhibition could impair NK cell function. NK cells from HD were treated in vitro with DMOG and then co-cultured with the K562 cell line. As expected, K562 killing by DMOG-treated NK-cells was significantly reduced demonstrating that the NK-cell cytotoxicity pathway was, at least in part, under the control of TET2 (*p* < 0.05, Fig. 5d).

### Hypomethylating agents rescue KIR expression on MDS NK cells

**Fig. 6:**
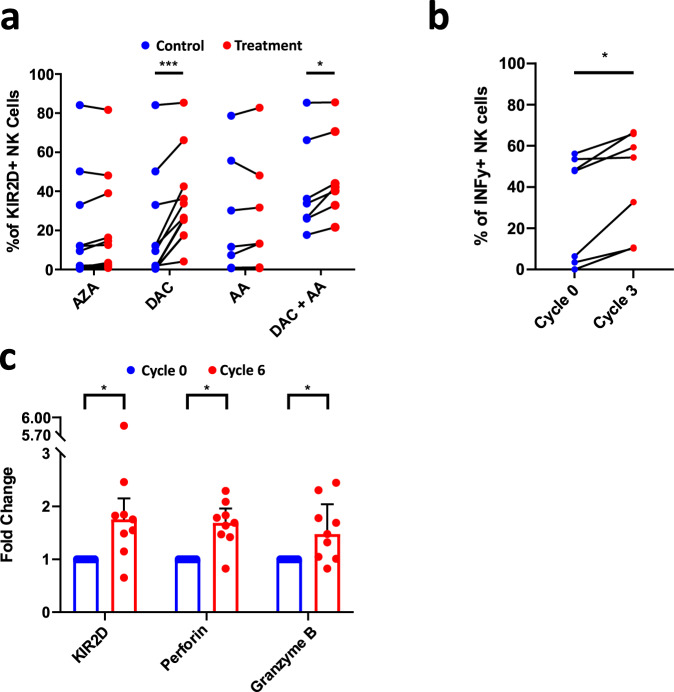
Hypomethylating agents normalize the NK cell phenotype of MDS patients. **a** Evaluation of KIR2D surface expression on NK cells of *TET2/IDH*^MUT^ patients after treatment with azacitidine (AZA, *n* = 12), decitabine (DAC, *n* = 12), acid ascorbic (AA, *n* = 7), and DAC+AA (*n* = 7). Nonparametric two-sided Wilcoxon matched-pairs signed rank test was used to determine statistical significance, DAC: ***p = 0.001, DAC+AA: *p = 0.0156. **b** NK cells were isolated from patients’ PBMC before and after 3 cycles of treatment with AZA, and cultured overnight at 100U/ml of IL-2. Subsequently, cells were cultured with PMA-Ionomycin for 6 h. The frequency of responding cells in terms of IFN-γ was assessed by flow cytometry (*n* = 7). **c** KIR2D, perforin, and granzyme B expression were measured by flow cytometry in the blood of MDS patients (*n* = 9) before (in blue) and after (in red) 6 cycles of AZA treatment. Nonparametric two-sided Wilcoxon matched-pairs signed rank test was used to determine statistical significance, KIR2D: *p = 0.0195, Perforin: *p = 0.0273, Granzyme B: *p = 0.0273. Data are presented as medians and interquartile ranges. Source data are provided as a Source Data file.

To evaluate whether demethylation could restore KIR expression on NK cells, PBMC from *TET2*^MUT^ patients were cultured in vitro in presence of IL-2 and with or without AZA or DAC for 5 days. Then, KIR2D expression on NK cells was assessed by flow cytometry (Fig. 6a). Whereas AZA showed only a trend, DAC significantly restored the KIR expression on treated NK cells (*p* = 0.07 and *p* < 0.001, respectively). Further, l-AA was used either alone or in combination with DAC to treat PBMC of *TET2*^MUT^ MDS/CMML patients. l-AA associated with DAC significantly increased KIR expression in NK cells compared with DAC alone (*p* < 0.05). Of note, NK cells from HD and *TET2*^WT^ patients also increased KIR expression in response to HMA with or without AA (Supplementary Fig. 8).

To confirm in vivo our observations, NK cells from MDS patients recruited in the clinical trial NCT02985190 were analyzed for their capacity to produce IFN-γ and to restore their phenotype, before and after 3 or 6 cycles of treatment with AZA. NCT02985190 clinical trial is a phase II study of efficacy and tolerance of AZA in MDS patients with concomitant systemic auto-immune and inflammatory disorders (SAID), for which the primary outcome measure was the overall response rate of MDS and SAID^29^. Functional experiments showed an increase in the production of INF-γ by NK cells stimulated in vitro by a mixture of PMA and ionomycin in patients after 3 cycles of treatment with AZA (*n* = 6, Fig. 6b). Moreover, KIR2D, perforin, and granzyme B expressions, quantified by flow-cytometry, were significantly increased in this group of patients after 6 cycles of treatment (*n* = 9, Fig. 6c). Importantly, despite the absence of significance due to the small number of patients, this effect of AZA on NK cell phenotype and function was similar in *TET2*^*WT*^ and *TET2*^*MUT*^ patients.

Taken as a whole, these findings showed that demethylation mediated by TET2 is pivotal in regulating NK cell biology, including the expression of KIR and functional proteins, and, more importantly, that treatment by HMA could normalize the hypermethylated profile harbored by NK cells in MDS patients.

## Discussion

In this work, we described the epigenetic, phenotypic, and functional consequences of *TET2* mutations for NK cells in MDS patients. We could demonstrate that mutations observed in MDS cancer cells, including in *TET2* and *IDH1/2* genes, are also generally observed in NK cells. Consequently, *TET2* mutations in NK cells are associated with perturbations in methylation of the genomic DNA inducing a reduced expression of KIRs, a locus on which TET2 directly binds, and of other key molecules for NK cell function including perforin and TNF-α. The reduced KIR expression and cytotoxicity of NK cells of healthy donors after TET2 inhibition confirmed its requirement for an efficient physiological NK cell function. Finally, NK cell phenotype can be restored in vitro and in vivo after treatment with ascorbic acid and HMA.

The fact that NK cell defects observed in MDS can be partly due to the oncogenic mutations at the origin of the disease could suggest that both events (i.e. mutations in MDS cells and NK cells) may synergize to sustain the emergence of myeloid malignancies. Abnormalities in NK cell phenotype and function could reduce the antitumor immuno-surveillance efficacy and allow the survival of potential pre-leukemic progenitors. Notably, NK-cell perturbations in MDS patients including reduction of activating NK receptors, such as NKp30, DNAM-1, and NKG2D, and impaired cytotoxicity, have already been reported^12,15,30^. KIR genotype has also been associated with clinical outcomes^31^. Our data suggest that these observations should be reinterpreted based on patient mutation profiles, especially for mutations associated with *TET2* or *IDH1/2* genes. In line with this comment, dissecting the specific role of TET2 in NK cells by contrast to other mutated genes commonly found in MDS or clonal hematopoiesis will require further investigations, which could be decisive in our comprehension of the disease and its treatments. Whether NK-cell function could be associated with a particular mutation landscape may have important consequences in developing NK-cell-based immunotherapeutic intervention. In that context, using autologous NK cells or attempting to rescue NK cell function in MDS patients may be impeded. Combining such therapeutic strategies with the use of HMA could provide a relevant option.

Loss-of-function somatic mutations in *TET2* are strongly associated with hematological malignancies, including myeloid, T- and B-cell malignancies and chronic lymphoproliferative disorders of NK cells (CLPD-NK)^32–34^. Germline mutations in *TET2* have been also associated with lymphoma^35,36^. Here, we found that *TET2* mutation in NK cells from MDS patients, with or without additional mutated genes, does not seem to affect NK cells from the oncogenic point of view (i.e. NK cells were not leukemic) but alters their phenotype and function. This raises the question of the requirement of other mutated genes, in addition to TET2, in order to induce malignant hemopathies and how these mutation profiles can be specific to lymphoid or myeloid malignancies.

Intriguingly, we noticed that *TET2* mutations in NK cells trended to increase methylation together on the gene body and its flanking regions, at least for genes involved in NK cell function. TET2 has been reported as preferentially targeting regulatory enhancers in hematopoietic and myeloid cells, notably to facilitate the recruitment of transcription factors^24,27^. However, gene bodies and other genomic regions can be also, although in a less efficient manner, targeted by TET2^24^. In addition, our results could suggest that key genes for NK cell function, including *KIR* and *PRF1*, are regulated in a coordinated manner by TET2 and more generally DNA methylation. If it is confirmed, this hypothesis would point out TET2 as a master regulator in NK cell development and education. It remains to be investigated whether and how genomic DNA methylation by TET2 is differentially regulated in NK cells, compared to myeloid subsets, due to differences in chromatin modification and accessibility or in the timing of TET2 expression during NK-cell lymphopoiesis.

From the mechanistic point of view, our results are reminiscent of the work by Wu et al. describing the role of the ascorbic acid in promoting KIR locus demethylation through the mobilization of Runx3, TET2, and TET3^22^. Interestingly, it has been recently shown that *TET2*^KO^ HSC treated with ascorbic acid could increase the 5hmC levels suggesting a role for TET proteins other than TET2 in this rescuing^26^. Herein, we showed that the addition of ascorbic acid to NK cells allows the restoration of KIR expression. Altogether, these data support the hypothesis that TET3 could compensate for *TET2* mutations in NK cells after treatment with ascorbic acid.

Clonal hematopoiesis (CH) is frequently presented as a precursor state for hematological malignancies, upstream of a continuum including MDS and then AML^37^. Arends et al. recently demonstrated that *TET2* mutations observed in individuals with CH could be found not only in myeloid lineage but also in B- and NK-cell^38^. Our results support the finding that CH infuses into the complete tree of hematopoiesis, including lymphopoiesis, and could also participate in reduced NK cell immunity in the elderly. It remains to discover the mechanisms by which *TET2* mutations alter the production of new lymphocytes in the elderly without significantly reducing the lymphocyte number, and with which consequences for the immune response. High-throughput experimental strategies, including multi-omics approaches combining RNAseq and ATACseq at the single cell level, applied not only to patients’ samples but also to *TET2*-edited HSC^26^ or iPSCs^36^, may help to solve this question.

Altogether, our findings showed that TET2 is a key epigenetic regulator of NK cells and that *TET2* mutations associated with MDS can alter their phenotype and function. In that context, HMA treatments could normalize the hypermethylated profile harbored by NK cells in MDS patients and may participate in the emergence of NK cell-mediated anti-tumor immunosurveillance.

## Methods

### Patient cohorts and samples

The first cohort was composed of 20 sternal bone marrow (BM) aspiration and 58 peripheral blood mononuclear cell (PBMC) samples that were collected from 63 MDS/CMML patients. Patients’ samples were obtained with written informed consent in the Hematology Departments of the Saint Louis Hospital, Paris, France (Supplementary Data 1 and Table 7). MDS and CMML diseases were defined according to the World Health Organization 2016 criteria^39^ and classified according to the Revised International Prognostic Scoring System (IPSS-R)^40^. Low-Risk (LR) disease was defined as IPSS-R very-low, low or intermediate whereas High-Risk (HR) disease was designated as IPSS-R high and very-high. Disease evolution was assessed according to the 2006 International Working Group criteria^41^. Among the 63 patients recruited, a few received treatments consisting of either Darbepoetin (EPO, *n* = 4) or Eltrombopag (TPO, *n* = 1) without obvious effect on immune cells, or the immunomodulating drugs Lenalidomide (*n* = 4) or the glucocorticoid Prednisone (*n* = 4). The second cohort was composed of MDS patients recruited in clinical trial #NCT02985190 (sponsored by Groupe Francophone des Myélodysplasies [GFM]) and repeatedly treated with azacitidine at 75 mg/m^2^/J subcutaneously daily for 7 days every 4 weeks^29^ (Supplementary Table 8). In addition, PBMC samples from 23 healthy individuals (HD) were collected through the EFS (Etablissement Français du Sang) (median age = 46 [27–66]). The study was approved by the Ethical Board Ile-de-France X and conducted in accordance with Helsinki’s declaration. Recruited patients gave their written informed consent for participating.

PBMCs were purified from BM and blood samples by using a ficoll-based gradient density method^13^ and stored in liquid nitrogen to be further analyzed.

### Patient KIR genotyping

Generic *KIR* typing was performed using a *KIR* multiplex PCR-SSP method as previously described^42^. The presence or absence of *KIR2DL1*, *2DL2*, *2DL3*, *2DL5*, *3DL1*, *2DS1*, *2DS2*, *2DS3*, *2DS4/1D*, *2DS5*, and *3DS1* genes were assigned. *KIR* genotypes were determined based on the presence or the absence of activating KIR. Thus, an AA *KIR* haplotype was defined by the presence of only *KIR2DS4* or its deleted variant (1D) as activating *KIR* gene, and a B+ (AB, BB) *KIR* haplotype by the presence of several activating KIR genes. Centromeric (AA, AB, BB) and telomeric (AA, AB, BB) *KIR* motifs were defined considering *KIR2DL2/3/S2* and *KIR3DL1/S1/2DS1/2DS4* genes for centromeric and telomeric motifs, respectively^43^ (Supplementary Table 3).

### Cell lines

HEK293T and Jurkat cell lines (ATCC) were maintained either with DMEM or RPMI1640 medium containing 10% of FBS, penicillin/streptomycin antibiotics, and l-glutamine.

For the measurement of target lysis, K562 cells were first cultured overnight in RPMI1640 without phenol red (Gibco) enriched with 10% FBS, non-essential amino acids (NEAA, Gibco), l-glutamine (Life Technologies) and Sodium Pyruvate (Eurobio). Prior killing assay, K562 cells were diluted at 1 × 10^6^/mL in a culture medium supplemented with 2.5 mM Probenecid (Invitrogen) and stained with calcein-AM (Invitrogen) for 30 min. Then, the excess calcein was washed out.

For in vitro cell treatment, PBMCs were resuspended in RPMI 1640 supplemented with 10% human serum (EFS, Etablissement Français du Sang), 1% Penicillin–Streptomycin (Dutscher), l-glutamine (Life Technologies), sodium pyruvate (EuroBio) and HEPES (EuroBio).

### Flow cytometry analysis

Frozen sternal BM aspiration and PBMCs were stained for 30 min on ice^13^, with the fixable viability dye eFluor 506 and monoclonal antibodies (mAb) listed in Supplementary Table 9. Of note, mAb used in this work to detect KIR molecules on the cell surface was able to identify KIR2DL1, KIR2DL2/DL3, KIR2DL4, KIR3DL1/DL2, KIR3DL1/DS1, KIR2DL5, and KIR2DS4 molecules. Cells were fixed in PBS with 2% of paraformaldehyde (PFA), acquired on a BD Fortessa X20 (flow cytometry core facility of Saint Louis Research Institute, Paris, France) (Supplementary Fig. 9). For intracellular staining, Foxp3 Transcription factor buffer set (eBioscence 00-5523-00) was used and data were acquired on a BD Canto II or Cytek Aurora flow cytometer. Data were analyzed using FlowJo v10.7 software or Cytek SpectroFlo v3.0.3.

### NK and T-cells isolation for mutational analysis and reduced representation bisulfite sequencing (RRBS)

After thawing, PBMCs were first labeled in PBS for 30 min at 4 °C with the fixable viability dye eFluor 506 (eBioscience) and then stained with antibodies specific for surface markers to distinguish NK cell subsets (at the whole or based on the surface expression of KIR2D and KIR3DL1/DL2 molecules) and T-cells, and directly sorted using BD Aria III system (Supplementary Table 9 and Supplementary Fig. 10). Dry pellets have been stored after cells sorting for DNA extraction. DNA was purified according to the manufacturer (DNeasy KIT, Qiagen) and quantified by Qubit (Thermo Fisher) for NGS and RRBS studies. Of note, clinical evaluation of MDS mutations was performed in the hematology laboratory, in St. Louis Hospital, Paris, on WMC obtained using gradient density separation on either BM or blood samples.

A panel of 80 genes commonly analyzed in myeloid malignancies (Supplementary Table 4) was examined using next-generation sequencing on an Illumina platform (Illumina, San Diego, CA, USA). Libraries were prepared from 200 ng of DNA using a custom probes panel for the capture of all coding sequences (SureSelectXT Target Enrichment System, Agilent, Santa Clara, CA, USA). Sequencing data were analyzed for variant calling using an in-house pipeline. High-probability pathogenic mutations were retained based on the available databases for SNPs, somatic mutations in cancer, prediction algorithms, and frequencies of variant read. Overall, a mean coverage of 851 reads/amplicon was achieved allowing reliable detection of low-burden mutations. Lolliplot has been realized using the St. Jude protein paint software available online at: https://proteinpaint.stjude.org/ (Fig. 3a)^44^. Major domains of TET2 protein were determined based on the work by Hu et al. ^45^. The mutational landscape was established using cBioPortal Cancer Genomics Portal OncoPrinter software available online at (https://www.cbioportal.org/) (Supplementary Fig. 6).

RRBS was performed by Diagenode (Seraing, Belgium) on dry samples of NK- and T-cells sorted from five MDS patients according to the manufacturer’s procedure. RRBS data are available on the Gene Expression Omnibus platform under the accession code GSE183020. 100 ng of purified genomic DNA was used, including pre-RRBS quality control, enzymatic digestion, and bisulfite conversion. Sequencing was performed in paired-end mode 50 bp (PE50), quality control of sequencing reads was performed using FastQC and reads alignment against human reference genome hg38/GRCh38 using Bismark v0.20.0 (available at https://www.bioinformatics.babraham.ac.uk/projects/bismark/) according to Diagenode protocol. Differential gene and site methylation analyses were performed by Genoplice Technology (Paris Biotech Santé, Paris, France). To assess the frequency of methylated CpG sites across all genes of a pathway of interest, gene lists were extracted from the KEGG pathway website^25^. The position of each CpG site was calculated relative to the transcript start and end positions as follows:

$${{{\rm {CG}}}}_{{{\rm {relative}}\,{{{{{\mathrm{position}}}}}}}}=\frac{{{{\rm {CG}}}}_{{{\rm {position}}}}-{{\rm {transcript}}}\,{{\rm {start}}}}{{{\rm {transcript}}}\,{{\rm {end}}}\,-{{\rm {transvript}}}\,{{\rm {start}}}}$$

Considering 10 kb upstream and downstream sites. Next, the frequency of methylation was calculated and aggregated across all genes of a given pathway for each sample.

Of note, all sequencing data were analyzed using the human reference genome hg38/GRCh38.

### Chromatin Immuno-Precipitation (ChIP)-qPCR assay

ChIP assay was performed with the Active Motif magnetic kit following the manufactory protocol. Briefly, after sonication, the sheared chromatin was incubated overnight with TET2 (Diagenode, C15410255), control H3 (Abcam, AB1791), and H3K18 (Abcam, AB1191) specific mAb and the isotype control (Diagenode, C15410206). ChIP-enriched DNA was analyzed by qPCR with SYBR Green Master Mix (Thermofisher). Primers are listed in Supplementary Table 10.

### Transfection and Luciferase assay

HEK293T cell lines (ATCC) were transfected by polyethyleneimine (PEI, Sigma) following previous publication^46^.

The plasmid pcDNA3-TET2 overexpressing the full-length mouse *Tet2* gene as the empty pcDNA3 vector was obtained from Addgene (Plasmid #60939 and #45346). All constructs have been verified by DNA sequencing.

One region of the predicted *KIR2DL1* promoter (KIRprom −147 + 60, Supplementary Fig. 11) was cloned in the pT109-tkLUC plasmid. 0.6 µg promoter vector together with the 2.7 µg TET2 plasmids were co-transfected in HEK293T cells with 0.06 µg of pRL-CMV Vector (#E2261 Promega) in presence of PEI solution.

In parallel, Jurkat cells (ATCC) were transfected with 0.26 µg of the KIR promoter plasmid and 0.026 µg of a pRL-CMV vector by Nucleofactor Kit V (Lonza). Jurkat transfected cells were then cultured at 1 × 10^6^/mL and treated with 500 µg of l-ascorbic acid (l-AA, Sigma) for 16 h before analysis^47^. The luciferase activity was measured after 24 h according to the manufacturer’s instructions (Dual-Luciferase Reporter Assay, Promega).

To investigate the role of the methylation in controlling the functional NK role, putative promoter regions, differentially methylated between KIR^HIGH^ and KIR^LOW^, were cloned into pCpGfree basic vector (Invivogen) for testing promoter activity. To determine the effect of methylation on the activity of the region, in vitro methylation of the constructs was performed with M.SssI (NEB). 0.6 µg promoter vector, “native” (unmethylated) or in vitro methylated, were co-transfected with 0.06 µg of pRL-CMV Vector (#E2261 Promega) in presence of PEI solution. After 24 h the Lucia and Firefly read-out was generated with a CLARIOstar^Plus^ (BMG LABTECH).

### Microfluidic multiplex qPCR

Multiplex qPCR analysis (Biomark, Fluidigm) was performed on 100 cells. NK cells as the whole and KIR^+^ (KIR2D or KIR3DL1/DS1 positive) or KIR^−^ (KIR2D or KIR3DL1/DS1 negative) NK cell subsets were sorted into 5 µL of reverse transcription/pre-amplification mix, prior to multiplex qRT-PCR following manufacturer’s protocol^48^. Briefly, the mix contained 2X Reaction mix and Superscript III (CellDirect One-Step qRT–PCR kit, Invitrogen) and 0.2X Taqman assay-specific probes (Life technologies). Targeted cDNA pre-amplification was performed for 19 cycles and the pre-amplified product was diluted 1:5 in TE buffer before processing with Dynamic Array protocol according to the manufacturer’s instructions (Fluidigm). All the probes used for this analysis are described in Supplementary Table 11. Mean expressions for *RPL27* and *GAPDH* housekeeping genes were used for signal normalization.

### In vitro cell treatment and functional assay

PBMC were treated in cell culture medium (i.e., RPMI 1640 supplemented with 10% fetal bovine serum) with 0.5 or 1 µM of AZA, DAC, dimethyloxaloylglycine (DMOG) (Sigma Aldrich), alone or in combination with 125 µM Ascorbic Acid. Interleukin-2 (IL-2) (Miltenyi Biotec) was added to a final concentration of 100 UI/mL. DMSO condition was used as a control. The treatments were maintained for 5 days with a half change of medium after 48 h (IL2 ± drugs or DMSO). Then, cells were collected and NK cells were analyzed for their phenotype (Supplementary Table 9 and Supplementary Fig. 9).

After treatment with DMOG, PBMCs were collected, enumerated, and co-cultured with calcein-labeled K562 cells in 96-well U bottom plates in a medium supplemented with Probenecid at 10:1 effector:target ratio. The killing was quantified after 4 h of incubation at 37 °C by measuring calcein-release into the supernatant. The specific killing was calculated as below: (Measured fluorescence of K562 + PBMC well—Spontaneous Fluorescence)/(Maximum fluorescence—Spontaneous fluorescence)*100.

For the ex vivo IFN-γ quantification, enriched NK cells from MDS patients enrolled in the NCT02985190 clinical trial^29^ were cultured with PMA-Ionomycin (SIGMA, 50 and 500 ng/mL, respectively) for 5 h at 37 °C. Brefeldin A (Sigma) was added at a final concentration of 10 µg/ml after 1 h of incubation. The percentage of IFN-γ positive cells was estimated by flow cytometry in the CD3^−^CD56^+^ NK cells. Spontaneous release was detected in the absence of target cells.

### Statistical analysis

Data are shown as median ± interquartile range, if not otherwise specified. Cytometry analyses were extracted from FlowJo v10.7 software and analyzed with Graph Pad Prism v8.0 software. Unpaired statistical analyses were calculated with the nonparametric Mann–Whitney test. Paired statistics analyses were calculated with the nonparametric Wilcoxon matched-pairs signed rank test. The Kolmogorov–Smirnov *D* test was used to calculate differences between treated/transfected and control situations. Pearson coefficient (*r*) was calculated to evaluate significant correlation. The receiver operation characteristic (ROC) curve was realized based on *TET2* mutation status and percentage of KIR2D + NK cells. The area under the curve, confidence interval, *p*-value, and likelihood ratio were calculated. A Friedman test followed by a Dunn’s test was used to calculate differences in experiments with multiple conditions. *p* < 0.05 was considered significant.

### Reporting summary

Further information on research design is available in the Nature Portfolio Reporting Summary linked to this article.

## Supplementary information

Supplementary Information

Description of Additional Supplementary Files

Supplementary Data 1

Supplementary Data 2

Supplementary Data 3

Reporting Summary

## Data Availability

Reduced representation bisulfite sequencing (RRBS) data that support the findings of this study have been deposited in the Gene Expression Omnibus data repository with the accession number GSE183020. The in vitro data generated in this study are available in the Supplementary information. Sequences of oligonucleotides used for the KIR genotyping (PCR-SSP) are limited to non-commercial and research use and are available upon request to Dr. K. Gendzekhadze (kgendzek@coh.org), City Of Hope Medical Foundation, 1500 Duarte Rd, Duarte, CA 91010. Source data are provided with this paper as a Source Data file. Source data are provided with this paper.

## Source data

Source Data
