# Supplementary material for: Myelodysplastic Syndrome associated TET2 mutations affect NK cell function and genome methylation
Source: Nat Commun. 2023 Feb 3;14:588. doi: 10.1038/s41467-023-36193-w (PMC9898569; doi:10.1038/s41467-023-36193-w)
Supplement: Supplementary file 1 — Supplementary Information [file 41467_2023_36193_MOESM1_ESM.pdf]

Myelodysplastic Syndrome associated TET2 mutations affect NK cell function and genome methylation

Supplementary Information

Supplementary Figure 1. KIR expression on $CD3^+$ T cells in MDS/CMML patients according to the presence or absence of *TET2/IDH* mutations.
Supplementary Figure 2. Expression of KIR2D, NKG2A and Perforin in blood NK cells without specific treatments.
Supplementary Figure 3. Percentages of NK cells positive for various KIR molecules in *TET2/IDH*^{WT} and *TET2/IDH*^{MUT} MDS patients.
Supplementary Figure 4. KIR and Perforin correlation in MDS/CMML patients with *TET2/IDH* mutations.
Supplementary Figure 5. Fraction of Perforin positive NK cells according to KIR expression.
Supplementary Figure 6. Mutational landscape in the MDS/CMML cohort.
Supplementary Figure 7. Percentage of VAF in the WMC, sorted NK/T cells and KIR^+ and KIR^- NK cells at diagnosis.
Supplementary Figure 8. Percentages of $KIR2D^+$ NK cells in healthy donors, *TET2*^{WT} and *TET2*^{MUT} MDS patients after *in vitro* treatment.
Supplementary Figure 9. NK cells phenotyping.
Supplementary Figure 10. Flow Chart.
Supplementary Figure 11. Analysis of KIR2DL1 proximal promoter and localization of primer used for CHIP and luciferase vector construction.

Supplementary Table 1. Circulating NK cells phenotyping.
Supplementary Table 2. Specific KIR Locus phenotyping and NKG2A in circulating NK cells.
Supplementary Table 3. KIR Locus genotyping on 22 MDS/CMML patients.
Supplementary Table 4. List of 80 genes for targeted Next-Generation Sequencing.
Supplementary Table 5. Mutations association in the whole cohort according to *TET2/IDH* mutations.
Supplementary Table 6. NGS at diagnosis in WMC and in NK/T sorted cells.
Supplementary Table 7. Patients' characteristics.
Supplementary Table 8. Patients of the Clinical Trial NCT02985190.
Supplementary Table 9. List of Antibodies used for Flow Cytometry.
Supplementary Table 10. List of primers used for the ChIP-qPCR assay.
Supplementary Table 11. Specificities and probes used in the Multiplex RT-PCR analysis.

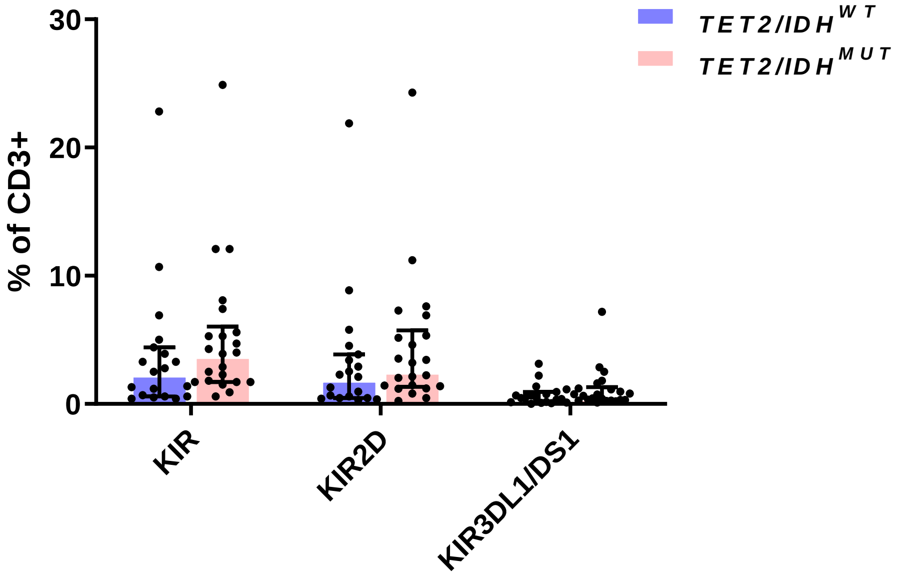

Supplementary Figure 1. KIR expression on CD3^{+} T cells in MDS/CMML patients according to the presence or absence of *TET2/IDH* mutations. Representation of the KIR, KIR2D and KIR3DL1/DS1 expression in blood CD3+ T cells of *TET2/IDH*^{WT} (n=19) and *TET2/IDH*^{MUT} (n=22). For all the analysis, median is represented, and error bars represents interquartile range. KIR = (KIR2D+ KIR3DL1/DS1-, KIR2D+ KIR3DL1/DS1+ and KIR2D- KIR3DL1/DS1+)

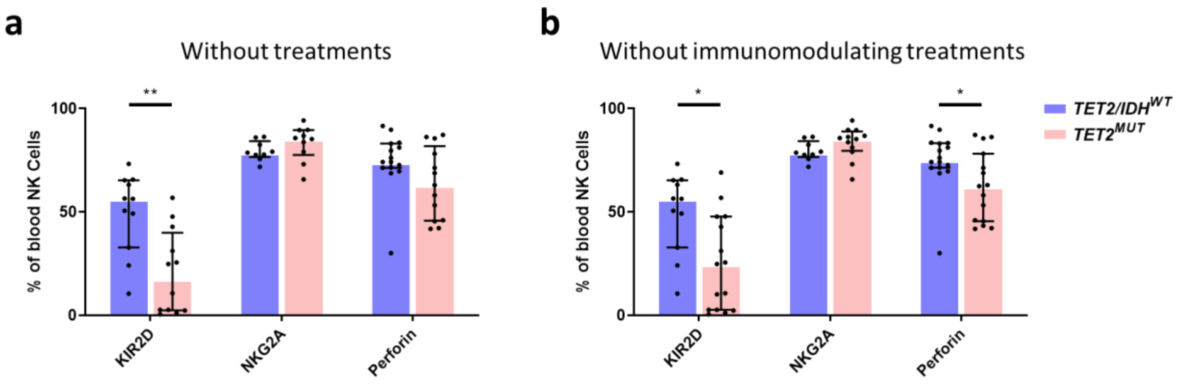

Supplementary Figure 2. Expression of KIR2D, NKG2A and Perforin in blood NK cells in absence of treatments. Expression of KIR2D, NKG2A and perforin in blood NK cells has been quantified by flow-cytometry. (a) markers expressions were analyzed in comparing *TET2/IDH*^{WT} (n=11 for KIR2D, n=9 for NKG2A n=15 for perforin) and *TET2*^{MUT} (n=13 for KIR2D, n=11 for NKG2A, n=13 for perforin) MDS patients in absence of any treatment. Statistics were calculated with the nonparametric Mann-Whitney test, two-sided, ** p=0.0036. (b) A similar analysis has been performed in *TET2/IDH*^{WT} (n=11 for KIR2D, n=9 for NKG2A, n=16 for perforin) or *TET2*^{MUT} (n=15, n=12 for NKG2A) MDS patients in absence of immunomodulating treatments. Data are presented as medians and interquartile ranges. Statistics were calculated with the two-sided nonparametric Mann-Whitney test, *p=0.0305, ** p=0.0092.

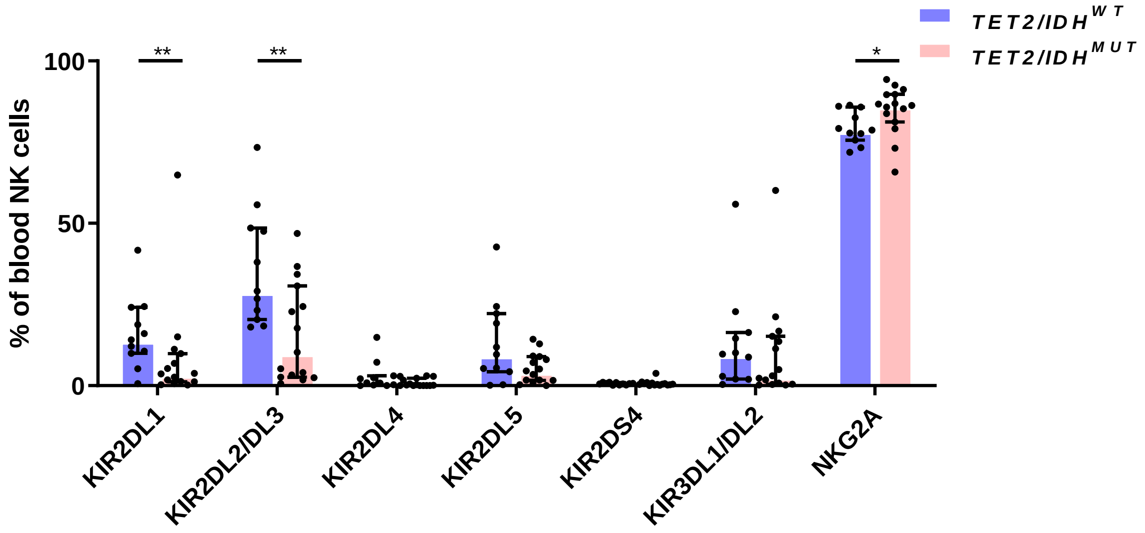

Supplementary Figure 3. Percentages of NK cells positive for various KIR molecules in *TET2/IDH*^{WT} and *TET2/IDH*^{MUT} MDS patients. *TET2/IDH*^{WT} (n= 11) are represented by blue bar and *TET2/IDH*^{MUT} (n=15) are represented by red bar. Data are presented as medians and interquartile ranges. Statistics were calculated with the two-sided nonparametric Mann-Whitney test. KIR2DL1: ** p=0.0092, KIR2DL2/L3: ** p=0.0077, NKG2A: * p=0.0246.

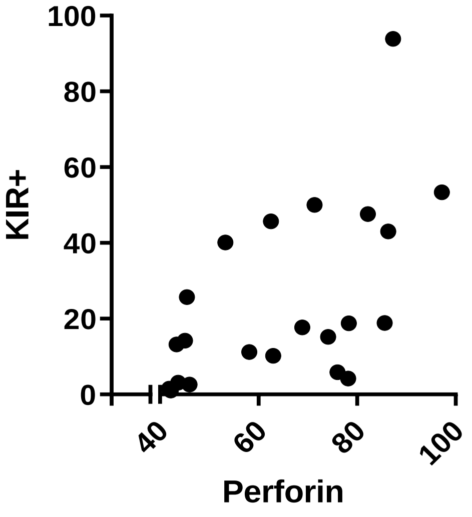

Supplementary Figure 4. KIR and Perforin correlation in MDS/CMML patients with *TET2/IDH* mutations. Correlation between the % of KIR (KIR2D+ KIR3DL1/DS1-, KIR2D+ KIR3DL1/DS1+ and KIR2D- KIR3DL1/DS1+) expressing NK cells and the % of Perforin expressing NK cells in *TET2/IDH*^{MUT} MDS/CMML patients (n=22). The nonparametric Spearman's rank-order correlation, two-sided, has been used, r =0.651 p=0.001.

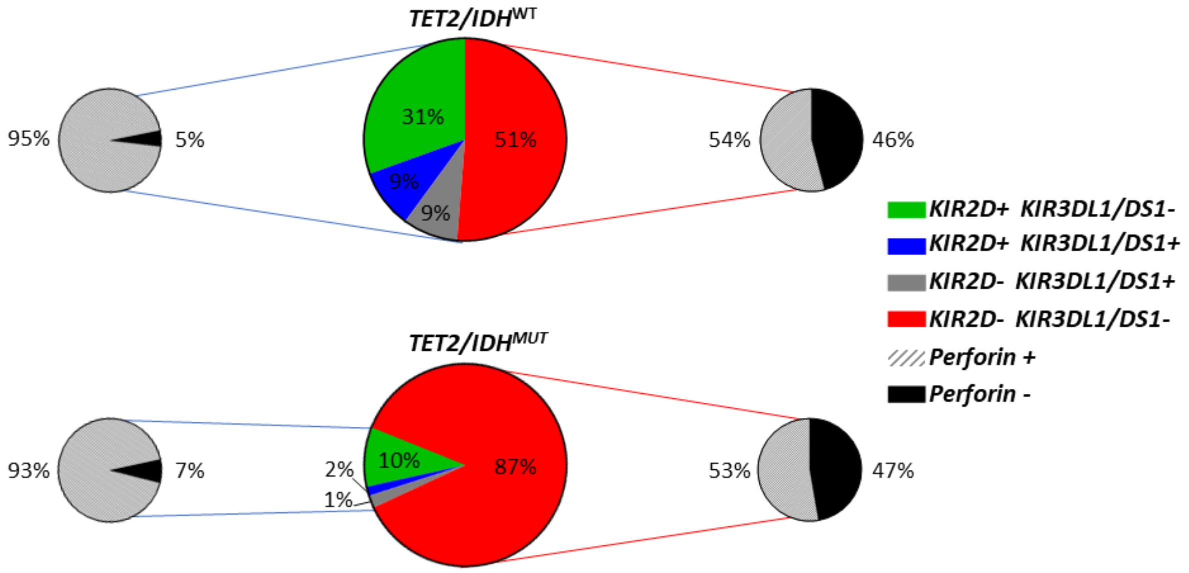

Supplementary Figure 5. Fraction of Perforin positive NK cells according to KIR expression. Central pie charts represent the expression of KIR molecules on blood NK cells in *TET2/IDH^{WT}* (n=19) and *TET2/IDH^{MUT}* (n=22). Lateral pie charts represent the percentage of Perforin positive and negative cells in KIR+ (left) and KIR- (right) NK cells. KIR (KIR2D+ KIR3DL1/DS1-, KIR2D+ KIR3DL1/DS1+ and KIR2D- KIR3DL1/DS1+)

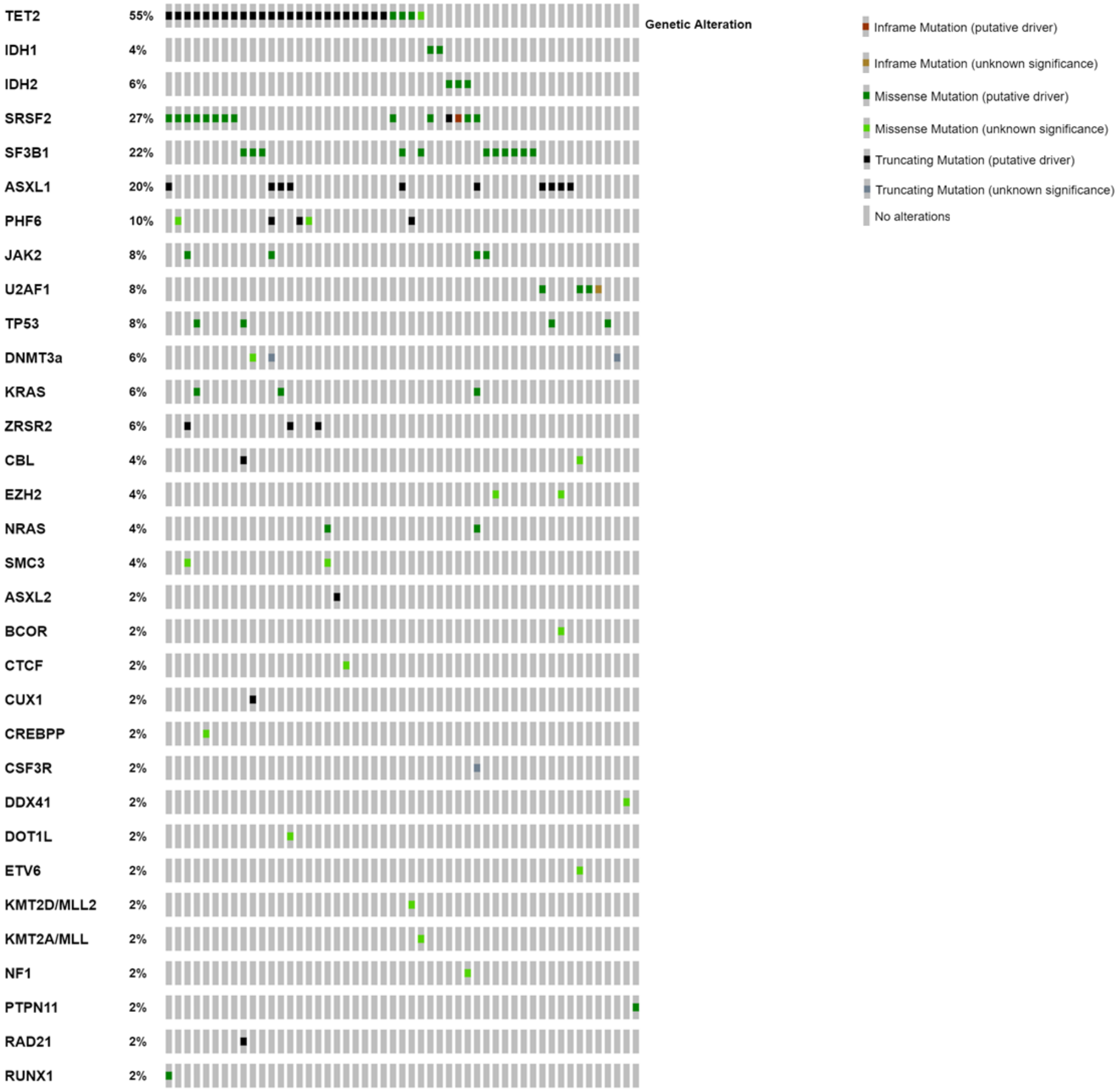

Supplementary Figure 6. Mutational landscape in the MDS/CMML cohort. Mutational Landscape of the 51 patients carrying mutation in at least one of the 80 genes analyzed by NGS.

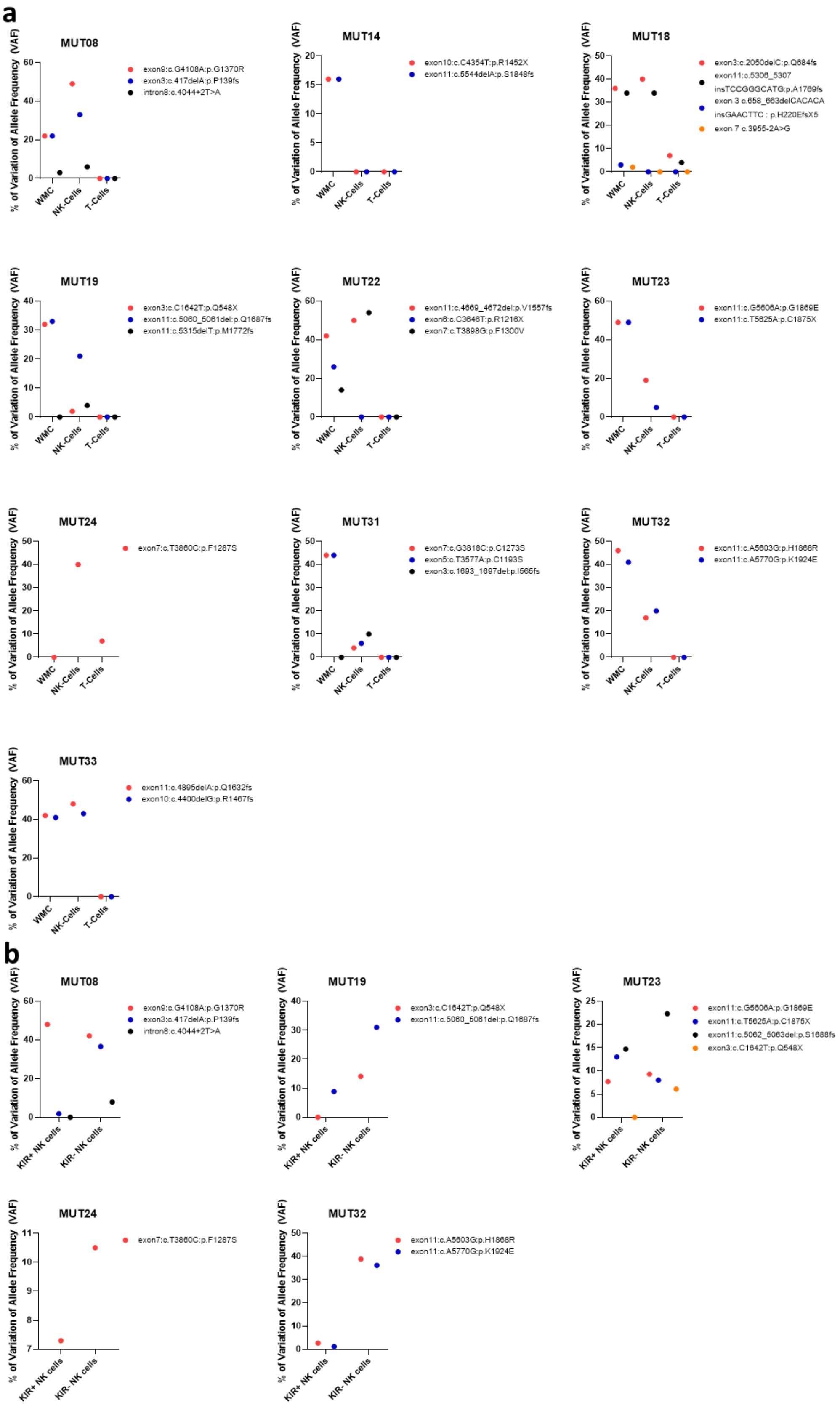

Supplementary Figure 7. Percentage of VAF in the WMC, sorted NK/T cells and KIR+ and KIR- NK cells at diagnosis. The percentage of Variant Allele Frequency (VAF) in the WMC and in sorted NK/T cells **(a)** or in KIR+ and KIR- NK cells **(b)** at diagnosis. WMC, White mononuclear cell.

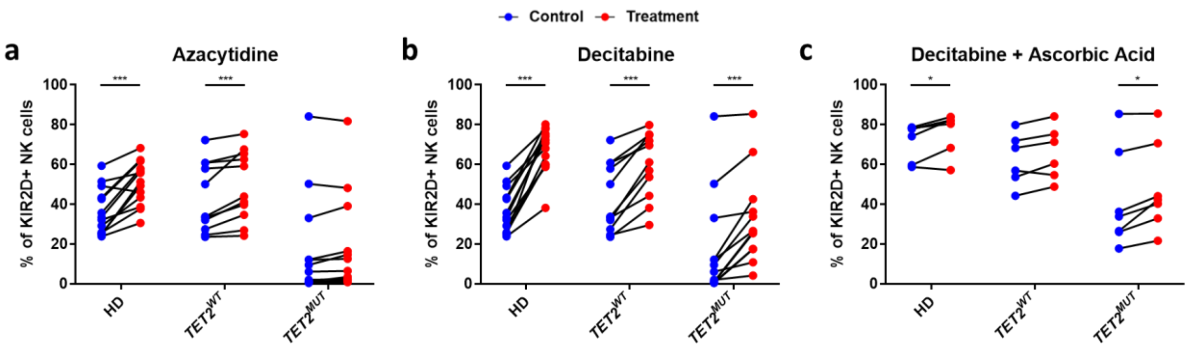

Supplementary Figure 8. Percentages of KIR2D+ NK cells in healthy donors, TET2^{WT} and TET2^{MUT} MDS patients after *in vitro* treatment. NK cells from healthy donors (n=13, n=7 for Decitabine + Acid Acorbic), *TET2*^{WT} (n=11, n=6 for Decitabine + Acid Ascorbic), and *TET2*^{MUT} (n=12, n=7 for Decitabine + Acid Ascorbic) MDS patients were treated 5 days with **(a)** Azacytidine (0.5µM), **(b)** Decitabine (0.5µM), and **(c)** Decitabine supplemented with Ascorbic Acid (125µM). Blue dots show the basal KIR2D expression in presence of DMSO for the test with Azacytidine or Decitabine alone, or in presence of Decitabine alone for the test with ascorbic acid. Red dots show KIR2D expression after the different treatments. Non-parametric two-sided Wilcoxon paired-test has been performed. Azacytidine treatment: ***p=0.005 for HD, and *** p=0.001 for *TET2*^{WT}. Decitabine treatment: *** p=0.0002 for HD, *** p=0.001 for *TET2*^{WT} and *** p=0.005 *TET2*^{MUT}. For Decitabine + Ascorbic Acid treatment: * p=0.0313 and * p=0.0156 for HD and *TET2*^{MUT} respectively.

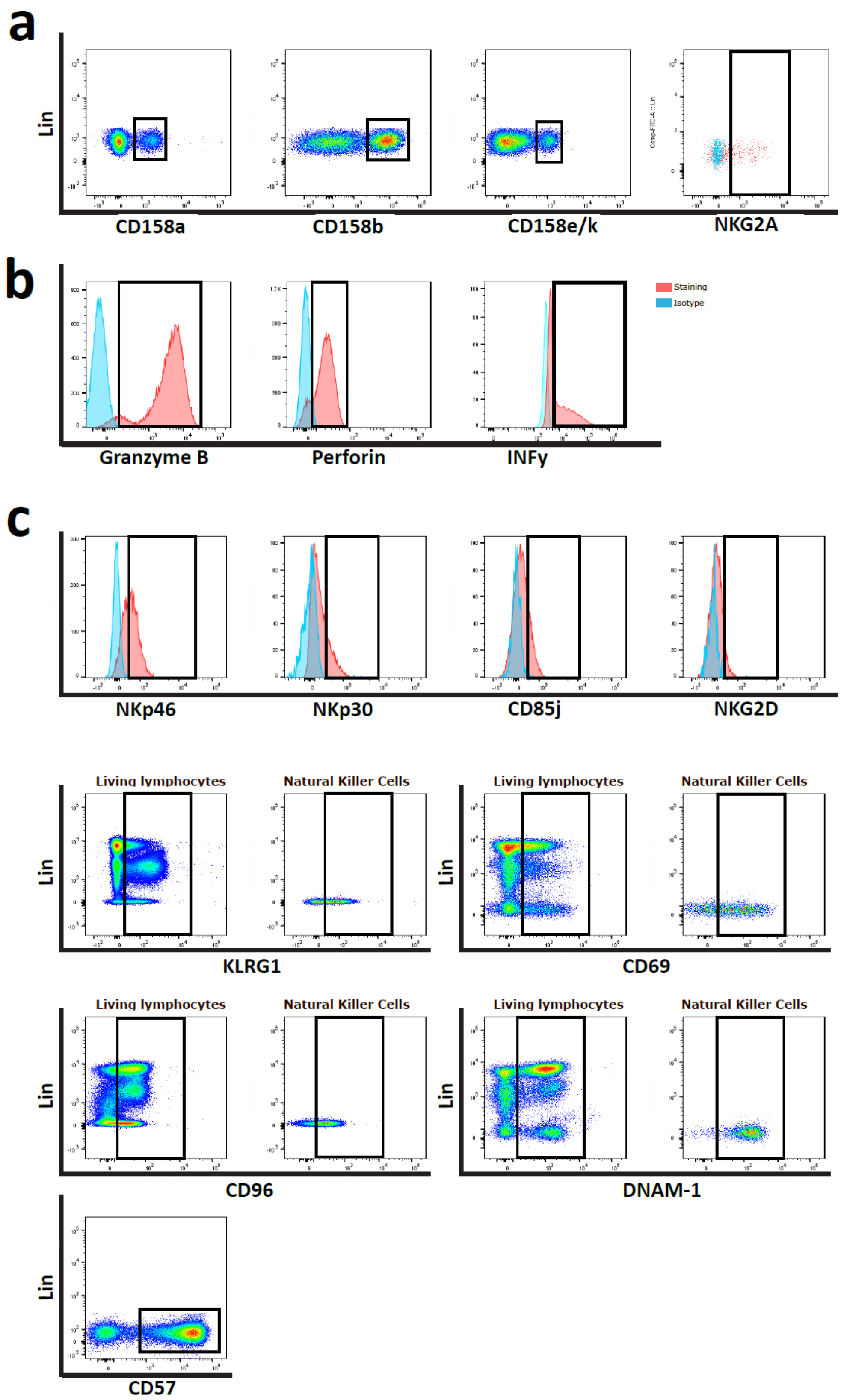

Supplementary Figure 9. NK cells phenotyping. Gating strategy of KIR phenotyping (a), Perforin/Granzyme B/IFN-γ (b) and general phenotype (c) of NK cells (Fig 1, 2C, 3B, 5D, 6).

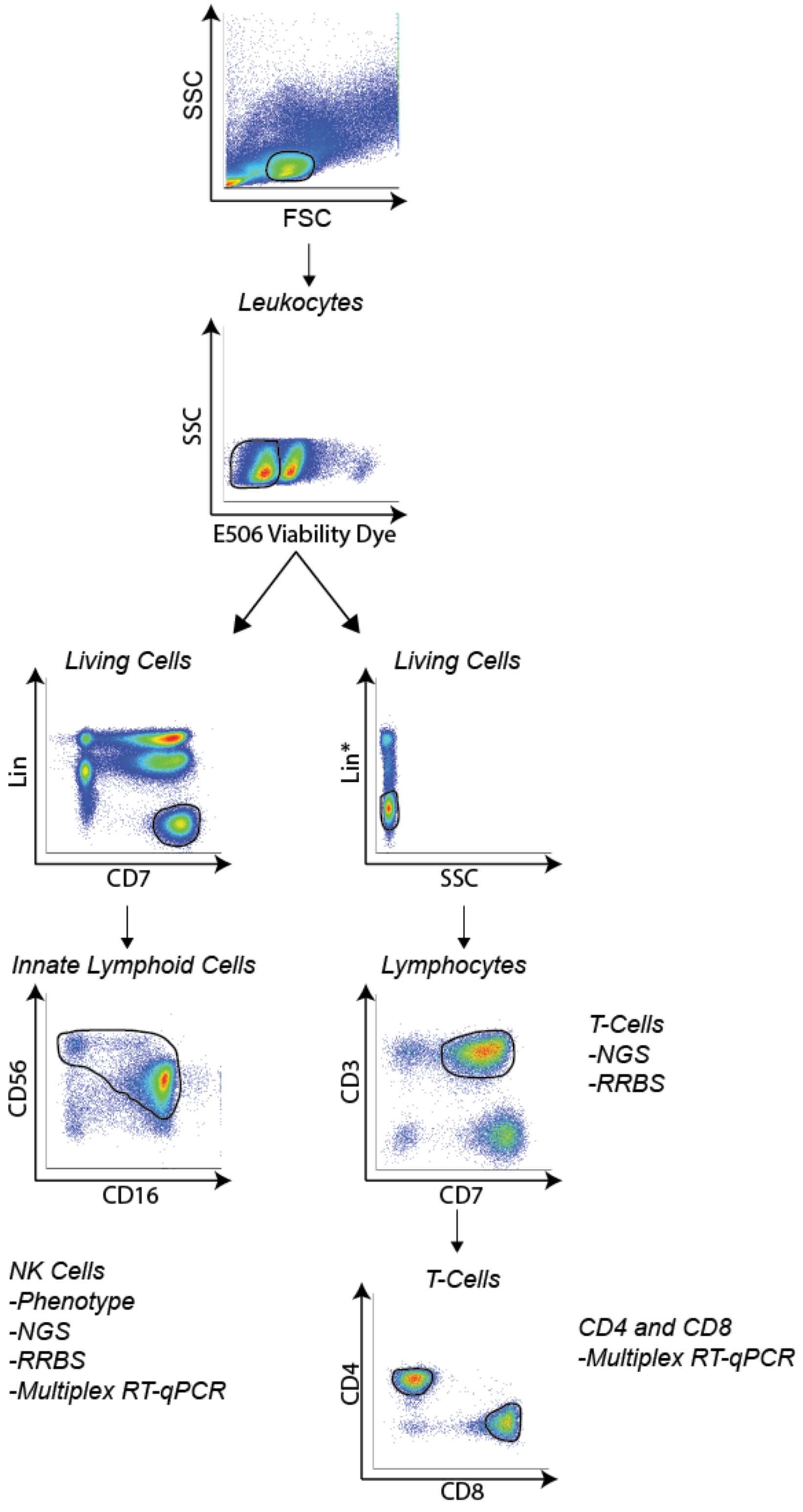

Supplementary Figure 10. Flow Chart. Flow cytometry chart deciphering the main gating strategy used to phenotype NK cells (Fig. 1, 2C, 3B, 5D, 6), or to sort NK and T cells (Fig 2A, 2B, 2D, 3F-H, 4, 5A-C).

Proximal promoter
TATA BOX -64 -58
KIR2DL1prom1 Primer F1 (-147 to -127) KIR2DL1prom2 Primer R1 (+43 to +63)
KIR2DL1prom2 Primer F2 (-63 to –43) KIR2DL1prom2 Primer R2 (+87 to +107)

```
TTGCTCTGTCGCCCAGGCTGGACTGCAGTGACACAATCTCAGCTCACTGCAACTTCTGCCTCCCAGGTTC
AAGCGATTCTCATGCCTCAGCCTCTTGAGTAGCTGGCGTTACAGGTGCCTGCCACCAGGCACGGCTAATT
TTTGGATTTTTAGCAGAGACACGGTTTCACTATATTGGCCAGGCTGCTCTCAAACTCCTTATCTCAGTTG
ATCCGCCCACCTCGGCTTCCCAACGTGCTGGGGAAACTTGATTTTCTATAGCATTATGTTACTGGATATT
TCTGTAAAATTTAAAATGAGGGAGGGAGAGAGACAGACGGAAAACAAACTCCAGAGTTGGGACTCTGGAA
TCTTGGGTCATGAGACAAATTTTAGATTAAACTACAAAACTCCAGAATTTACAGGTGGGGTTTTTACTGA
TAAAGTACAATTCTAAGATTGTAAATAATTGCATAATCCTTCCCTGGGAATTTAAATCATTTTAACTGGT
TCTGCTGTAATACTAGAAATACAAGCATGAAAAATTCTAATGGTTTATTAGTGACAATGACTCTGAAAAC
ATTAATAATACCTATTAGATATTTTGCATATTACACAGGAAGAAGAGTTTGAATCTCAGATAAAAACAAT
AGAAATACATGAAAAGTCTTTCATGTTAGCACAGATTTTAGGCATCTCGTGTTCGGGAGGTTGGATCTCA
GACGTGTTTTGAGTTGGTCATAGTGAAGGACACTAGGTGTCAAATTCTAGCGAGAACAATTTCCAGGAAG
CCGTGTTCCGCTCTTGAGCGAGCACCCACTGGGCCTCATGCAAGGTAGAAAGAGCCTGCGTACGTCACCC
TCCCATGATGTGGTCAACATGTAAACTGCATGGGCAGGGCGCCAAATAACATCCTGTGCGCTGCTGAGCT
GAGCTCGGTCGCGGCTGCCTGTCTGCTCCGGCAGCACCATGTCGCTCTTGGTCGTCAGCATGGCGTGTGT
TGGTGAGTCCTGGAAAGCAATAGAGGGAGGGAGTGAGGGGATGGAGATCTGGGCCCAGAGGTGGAGATAT
AGGCCTGGAGGTGGAGTTATGGGCCTGGAGTGGAGATCTGGGCCTGGAGTGGATATATGGGCCTAGAGAT
GGAGTGATGGGCCTAGAAGTGGAGATCTGGGCCCAGAGGTCGAGATATAGGCCTGGAGGTGGAGTGATGG
GACTGTAGTGGAGATCTGGGCCTGGAGTGGAGATAGGAACCTGGAGGGGAGATAGGAACCTGGAGGGGAG
ATATGGGCCTGGAGGTGGAGATATGGGCCTGGAGTGGAGTCATGGGCCTGGAGGTGGAGTTATGGGCCTG
CAGTAGAGATATGGGCCTGAAGTGGAGACATGGGCCTGGAGTGGAGATATGGGCCAGGAGTGGAGATATG
GGCCTAGAGGTCGATATCTGGGCCTGGAGTGGAGATATGGGCCAGGAGTGGAGATATGGGCCTAGAGGTC
GATATCTGGGCCTGGAGAGGAGATATGTGCCTAGGATGGAGATACGGGCCTGGGTGTGGAGATATGGGAC
TGGAGAGGATATATGGGCCTGGAGTGGAGATATGGGACTGGAGAGGAGATATGGACCTGGAGTGGAGATA
AGGGCCTGGATTGGAGATATGGGCCCAGGGTGGAGATCTGAGCCTGGATTGGAGATATGGGCCTGGATTG
GCGATATGGGCTTAGGGTGGAAATATCGGCCTGGAGTGGAGATATGGGCCTGGAGTGGAGATATGGGCTT
GAGGTGGGGATATGGACCTGGAGGCTGGGTCTCTGCACAGCCGACAGCCCTGTTCTTGGGTGCAGGTAGG
CACTGAGGGTGAGTTTACCTTCAGCCCAGGAAGGGCCTGGCTACCAAGACTCACAGCCCAGTGGGGGCAG
CAAGGGTGCCCTGGTTTGCCTGCAGATGGGTCATCCATCATGATCTTTCCCTCCCTCTATTGCTTTCCAG
GGTTCTTCTTGCTGCAGGGGGCCTGGCCACATGAGGGTGAGTCCTTCTCCCAACCTTCGGGTGTCATCTC
CCCACATAAGAGGATTTTCCTGAAATGGGAGGGAAGTCCTGTCAGGGAGTCTCTCATAAACTAGGAAGAA
GGGACCCTGGGGTGCTGGGCCCACATTTCTGACCTTGCCTCCCTGGCCTTTCATTCCCTTGGCAGAGTCA
AGTTCTGTGGGGACCAGGGTTAGACTACGGTGCTCAAAGCTGGGGTGTGTGGTGGGGAAGTGGTAGGAAC
AGCAGATCCTCTGAGGACAAAGGTGTTACTCACACACTTCAGCGTTTCCATGACGGTAGGGGCTGCAGTG
TGGCTGCTGTCATTCTACCAGAAGAGGTGGGAAAACCACAGCCATGGCCCTGACATTCCAATCCTCTGAT
GGGGACTCAGTTGTTTATTTTCGTTCAGGCATCGGCTGATATTCCATTCTCAAAGGACATGCCCTCCACC
CCATGTCTACCCTGTGTTGTTTTATGTGAGTAATCTTACAGTATTAAAATCTAGTAGGAGTCTCTTACTC
AGCACTTGCTCAAAGTTCTCAGCTGACACTTTTGTTGTAGGGAGACACCTTGTGTTTGCGGGATGGGTCC
TTCCTTTAGCCCTGGGCACCAAGGTGTGATAGCAGCCATAGAAACTTGGAAAGCGAGGAGAATCTTCAGA
GCACAGGGAGGGAGGGGCGGCTCCACATCCTCCTCTCTAAGGCGGTGCCTCCTTCTCCCCACGGTGGTCA
GGACAAGCCCTTGCTGTCTGCCTGGCCAAGCCCTGTGGTGCCTCCAGGACATGTGATTCTTCAGTGTCAT
TCTTATCTTGGGTTTAACAACTTCAGTCTGTAAAAGGAAGATGGGGTGCCTGTCCCTGAGCTCTACAACA
TAATATTCTGGAACAGCCTTTTCATGGGCCCTGTGACCCCAGCACACGCAGGGACCTATACATGTCGGGG
TTCACAACCACACTACCCCAGTGGGTGGTCGGCACCCAGCAACCCCCTGGAGATCACGGTCACAGGTCAG
AGGGCTCCTGTCTGGGATTCTCCTTGTCCCACCTCCTGAATCCCAGAGCTCCTGGTGGGCGTGTCCTTGC
GGGTCCCATCATGCAAGTCCTGACTGTATTTGGGGTAAAGGGGGATTGAATACAGGGAAATGGGTGCTGT
GGTGGGAAGAATAATTGTCCCCAGTGATGACTACATTCTAATCCCTGGAGTCTGTGACTATTTATGATAT
AGGGGAAGGGACTGAAGGAGAAGATGGAGCTCAGGTTGTTGATGAGTTGACCTTGAGATGGGGAGACAGC
CTGGACTGTCCTGATGGGCTCAGTGTAGTCACAGGGGTCCACAGGAAAGGAGGAGGAAGAGGGGAGTGGG
GATTACAGCAGCATAATGGGAGTCTCCATCAGCTTTGAAGGTGGAGGAAGTCCAGGAGCCATGAATGCAG
GTGGCCTATAGAGGCTGGAAAAGTCAAGGAACTGATTCTCCTGAGTCTCCAGAGGGAACGAAGCCCTGCA
GGTACCTTGATTTTACCCACGACAAACAGGGTCCGATTTCTGTCTCCAGAATTGGAAGGGGTTAGTGTGC
TCTCTCCTGCTGCCATGCTTCTGATAATTTTCTACAGCAGCAACAGGAAACCAACACTGGAACCCAGGTC
AAGGACAAGTTAAGAAACAACACAAGGATAGCCAGGCATGGTGGCAGGTGCATGTAATCCTAGCGACTTG
GGAGGCTGAGGGCAGGAGAATCACTTGAACCCAGGAGACAGAGGTTGCAGTAAGCCTAGACCACACCACT
TCACTCCAGCCTGGGCAAAGGAGTGAGACTCTGTCGCCAAAATTAATTAATTAATTAAAGAAACCAAACA
AGGAGAAGGTTGGC
```

Supplementary Figure 11. Analysis of KIR2DL1 proximal promoter and localization of primer used for CHIP and luciferase vector construction.

Supplementary Table 1. Circulating NK cells phenotyping.

	HD (n=23)	MDS/CMML (n=33)	p=	*TET2/IDH* ^{WT} (n=13)	*TET2/IDH* ^{MUT} (n=19)	p=
	Median [range] %	Median [range] %		Median [range] %	Median [range] %	
**NK cells**						
DNAM-1	96.2 [37.8-99.8]	90.5 [14.7-99.2]	0.0109	94.4 [14.7-99.2]	87.8 [33.7-98.8]	
NKG2D	65.4 [33.5-85.8]	61.4 [29-90.5]		62.9 [39.9-87.4]	59.9 [29-90.5]	
NKp30	78.6 [33.6-95.5]	79.5 [31.6-99.5]		81.1 [62-99]	74.5 [31.6-99.5]	
NKp46	33.2 [9.04-65.5]	37 [11.2-93.5]	0.0483	39.2 [15.6-93.5]	34.7 [11.2-90.3]	
CD85j	24.9 [5.56-76.5]	25.6 [11.7-76.5]		23.4 [11.7-56.9]	29.3 [13.7-76.5]	
CD96	5.51 [0.95-21.8]	11 [2.8-42.9]	0.0006	9.06 [5.67-42.9]	11.2 [2.8-41.4]	
KIR2D	54.8 [33.3-76.8]	31.2 [0.58-77.7]	0.0034	56.5 [10.5-77.7]	11.9 [0.58-69.1]	0.0005
CD57	63.2 [28.1-81]	60.1 [4.87-95.1]		63.1 [18.8-95.1]	58.9[4.87-85.6]	
KLRG1	21.9 [9.2-73.3]	29 [2.56-96.5]		26 [6.7-76]	38.5 [2.56-96.5	
CD69	20.9 [8.69-81.7]	24.8 [3.85-82.7]		47.7 [4.01-82.7]	23.6 [3.85-73.5]	
**CD56** ^{Dim} **NK cells**						
DNAM-1	96.1 [39.3-99.8]	87.9 [13.9-99.2]	0.0038	94.5 [13.9-99.2]	86.6 [33.7-98.7]	
NKG2D	66 [33.8-86.7]	61.3 [29.1-89.9]		62.3 [39.9-87.3]	60.6 [29.1-89.9]	
NKp30	68 [33.8-96.3]	79.4 [29.6-99.8]		83.6 [59.9-99]	73.7 [31-99.8]	
NKp46	28.5 [8.07-56.6]	35.1 [10.5-93.1]	0.0493	38.4 [10.7-93.1]	34.4 [10.5-89.9]	
CD85j	21.4 [5.72-75]	26.1 [11-76.9]		23.4 [11-57.1]	28.8 [14-76.9]	
CD96	4.32 [0.65-23.2]	10.9 [2.66-42]	0.0001	8.77 [4.53-37,5]	11.5 [2,66-41.6]	
KIR2D	60.8 [37-80]	35.1 [0.63-82.3]	0.0009	60 [10.7-82.3]	17.9 [0.63-71.2]	0.0004
CD57	67 [37.2-84.5]	64.1 [5.76-86.5]		69.6 [20.1-95.9]	60.7 [5.76-86.5]	
KLRG1	30.1 [9.54-79.2]	35.1 [2.31-98.3]		27.5 [6.85-80.1]	41.7 [2.31-98.3]	
CD69	20 [8.88-84.9]	25.4 [3.8-83.1]		49.6 [3.8-83.1]	23.8 [4.13-76]	
**CD56** ^{Bright} **NK cells**						
DNAM-1	97.5 [8.26-99.9]	90.4 [21.2-99.6]	0.0037	92.3 [21.2-99.6]	89 [33.7-96.6]	
NKG2D	64.6 [19.9-96.8]	83.1 [19.7-97.7]	0.0046	88.9 [58.4-95.1]	79.2 [19.7-97.7]	
NKp30	63 [20.3-93.8]	81.2 [29-98.5]	0.0029	79 [45.3-98.5]	84.4 [29-97.1]	
NKp46	62.1 [10.7-85.5]	64.5 [34-99.1]		63.9 [39.7-98.5]	64.5 [34-99.1]	
CD85j	24.7 [2.33-82.1]	25.7 [12.1-68.1]		22.4 [13.8-64]	31 [12.1-68.1]	
CD96	11 [3.28-29.6]	17.4 [2.48-54.5]	0.0182	33.5 [14.7-54.5]	15.2 [2.48-42.6]	0.0045
KIR2D	6.27 [3.07-17.8]	6.55 [0-40.9]		14.1 [3.34-40.9]	1.76 [0-26.2]	0.0035
CD57	3.51 [0.21-38.1]	3.8 [0.18-83.1]		3.64 [1.52-83.1]	4.58 [0.18-47.7]	
KLRG1	7.2 [2.33-26.8]	15.8 [3.16-78.3]	0.0012	10.2 [4.97-42.1]	20.4 [3.16-78.3]	
CD69	5.43 [1.72-35.1]	19.7 [2.34-78.5]	<0.0001	17.9 [6.05-78.5]	22.3 [2.34-66.1]	

Unpaired statistics analysis has been realized by using nonparametric Mann Whitney test two sided.

Supplementary Table 2. Specific KIR Locus phenotyping and NKG2A in circulating NK cells.

	TET2/IDH^{WT} (n=11)	*TET2/IDH*^{MUT} (n=15)	p=
	**Median [range] %**	**Median [range] %**	
**KIR2DL1**	14.1 [0.65-41.7]	3.63 [0.2-64.9]	0.0092
**KIR2DL2/DL3**	29.1 [18-73.4]	10.3 [0.62-46.9]	0.0077
**KIR3DL1/DL2**	9.71 [0.34-55.9]	3.08 [0.18-60.1]	
**NKG2A**	78.7 [71.9-86.4]	86.3 [65.8-9.3]	0.0246

Unpaired statistics analysis has been realized by using nonparametric Mann Whitney test two sided.

Supplementary Table 3. KIR Locus genotyping on 22 MDS/CMML patients

Identifiant	2DL1	2DL2	2DL3	2DL4	2DL5	3DL1	3DL2	3DL3	2DS1	2DS2	2DS3	2DS4	1D (2DS4v)	2DS5	3DS1	Genotype KIR
MUT01	+	-	+	+	-	+	+	+	-	-	-	-	+	-	-	AA
WT03	+	+	+	+	-	+	+	+	-	+	-	-	+	-	-	AB
WT04	+	+	+	+	-	+	+	+	-	+	-	+	+	-	-	AB
WT05	+	+	+	+	+	+	+	+	+	+	+	-	+	+	+	AB
MUT07	+	+	+	+	+	+	+	+	-	+	+	+	+	-	-	AB
WT08	+	+	+	+	-	+	+	+	-	+	-	+	+	-	-	AB
MUT08	+	+	+	+	-	+	+	+	-	+	-	-	+	-	-	AB
MUT14	+	-	+	+	-	+	+	+	-	-	-	+	+	-	-	AA
MUT15	+	+	+	+	+	+	+	+	+	+	-	+	-	+	+	AB
MUT16	+	+	+	+	-	+	+	+	-	+	-	+	+	-	-	AB
WT13	+	-	+	+	-	+	+	+	-	-	-	-	+	-	-	AA
WT14	+	+	+	+	+	+	+	+	-	+	+	-	+	-	-	AB
WT15	+	-	+	+	+	+	+	+	+	-	-	+	-	+	+	AB
WT17	+	+	+	+	-	+	+	+	-	+	-	-	+	-	-	AB
WT18	+	-	+	+	-	+	+	+	-	-	-	+	+	-	-	AA
MUT23	+	-	+	+	+	+	+	+	+	-	-	-	+	+	+	AB
MUT24	+	-	+	+	-	+	+	+	-	-	-	+	+	-	-	AA
WT22	+	-	+	+	-	+	+	+	-	-	-	+	+	-	-	AA
WT24	+	+	+	+	+	+	+	+	+	+	-	+	-	+	+	AB
MUT27	+	+	+	+	-	+	+	+	-	+	-	-	+	-	-	AB
MUT30	+	+	+	+	+	+	+	+	+	+	+	-	+	-	+	AB
WT29	+	+	+	+	-	+	+	+	-	+	-	+	+	-	-	AB

Supplementary Table 4. List of 80 genes for targeted Next-Generation Sequencing.

N°	Gene	Reference	N°	Gene	Reference
1	*ASXL1*	NM_015338	41	*KMT2A/MLL*	NM_001197104
2	*ASXL2*	NM_018263	42	*KMT2D/MLL2*	NM_003482
3	*ATM*	NM_000051	43	*KRAS*	NM_033360
4	*ATRX*	NM_000489	44	*LIG4*	NM_002312.3
5	*BCOR*	NM_017745	45	*MDM4*	NM_002393
6	*BCORL1*	NM_021946	46	*MECOM*	NM_001105078
7	*BRAF*	NM_004333	47	*MPL*	NM_005373
8	*BRCA1*	NM_007294	48	*MYC*	NM_002467
9	*BRCA2*	NM_000059	49	*NF1*	NM_001042492
10	*BRCC3*	NM_024332	50	*NPM1*	NM_002520
11	*CALR*	NM_004343	51	*NRAS*	NM_002524
12	*CBL*	NM_005188	52	*PARN*	NM_002582
13	*CDAN1*	NM_138477	53	*PHF6*	NM_001015877
14	*CEBPA*	NM_004364	54	*PPM1D*	NM_003620
15	*CHEK2*	NM_007194.3	55	*PRPF8*	NM_006445
16	*CREBBP*	NM_004380	56	*PTEN*	NM_00314
17	*CSF3R*	NM_156039	57	*PTPN11*	NM_002834
18	*CTC1*	NM_02599	58	*RAD21*	NM_006265
19	*CTCF*	NM_006565	59	*RIT1*	NM_006912
20	*CUX1*	NM_001913	60	*RPL11*	NM_000975
21	*DDX41*	NM_016222	61	*RPL5*	NM_000969
22	*DKC1*	NM_0001363.2	62	*RTEL1*	NM_032957
23	*DNAJC21*	NM_001012339	63	*RUNX1*	NM_001754
24	*DNMT3A*	NM_022552	64	*SAMD9*	NM_017654
25	*EP300*	NM_001429	65	*SAMD9L*	NM_152703
26	*ERCC6L2*	NM_001010895	66	*SBDS*	NM_016038
27	*ETNK1*	NM_018638	67	*SETBP1*	NM_015559
28	*ETV6*	NM_001987	68	*SF1*	NM_004630
29	*EZH2*	NM_004456	69	*SF3B1*	NM_012433
30	*FLT3*	NM_004119	70	*SMC1A*	NM_006306
31	*GATA2*	NM_032638	71	*SMC3*	NM_005445
32	*GSKIP*	NM_001271904	72	*SRP72*	NM_006947
33	*HRAS*	NM_005343	73	*SRSF2*	NM_003016
34	*IDH1*	NM_005896	74	*STAG2*	NM_001042749
35	*IDH2*	NM_002168	75	*TET2*	NM_001127208
36	*IRF1*	NM_002198	76	*TP53*	NM_001126112
37	*JAK2*	NM_004972	77	*U2AF1*	NM_006758
38	*KDM5A*	NM_001042603	78	*U2AF2*	NM_007279
39	*KDM6A*	NM_021140	79	*WT1*	NM_024426
40	*KIT*	NM_000222	80	*ZRSR2*	NM_005089

Supplementary Table 5. Mutations association in the whole cohort according to *TET2/IDH* mutations.

	All patients (n = 63)		*TET2* mutation profile		
		IDH1/2 ^{MUT} patients (n = 28)	*TET2* ^{MUT} patients (n = 28)	*TET2/IDH* ^{WT} patients (n = 30)	p=
ASXL1	10	0	5	5	NS
ASXL2	1	0	1	0	NS
ATM	0	0	0	0	NA
ATRX	0	0	0	0	NA
BCOR	1	0	0	1	NS
BCORL1	0	0	0	0	NA
BRAF	0	0	0	0	NA
BRCA1	0	0	0	0	NA
BRCA2	0	0	0	0	NA
BRCC3	1	0	1	0	NS
CALR	0	0	0	0	NA
CBL	2	0	1	1	NS
CDAN1	0	0	0	0	NA
CEBPA	0	0	0	0	NA
CHEK2	0	0	0	0	NA
CREBBP	1	0	1	0	NS
CSF3R	1	0	0	1	NS
CTC1	0	0	0	0	NA
CTCF	1	0	1	0	NS
CUX1	2	0	2	0	NS
DDX41	1	0	0	1	NS
DKC1	0	0	0	0	NA
DNAJC21	0	0	0	0	NA
DNMT3A	3	0	2	1	NS
DOT1L	1	0	1	0	NS
EP300	0	0	0	0	NA
ERCC6L2	0	0	0	0	NA
ETNK1	0	0	0	0	NA
ETV6	1	0	0	1	NS
EZH2	2	0	0	2	NS
FLT3	0	0	0	0	NA
GATA2	0	0	0	0	NA
GSKIP	0	0	0	0	NA
HRAS	0	0	0	0	NA
IDH1	2	2	0	0	NS
IDH2	3	3	0	0	NS
IRF1	0	0	0	0	NA
JAK2	4	0	2	2	NS
KDM5A	0	0	0	0	NA
KDM6A	0	0	0	0	NA
KIT	0	0	0	0	NA
KMT2A/MLL	1	0	1	0	NS
KMT2D/MLL2	1	0	1	0	NS
KRAS	3	0	2	1	NS
LIG4	0	0	0	0	NA
MDM4	0	0	0	0	NA
MECOM	0	0	0	0	NA
MPL	0	0	0	0	NA
MYC	0	0	0	0	NA
NF1	1	1	0	0	NA
NPM1	0	0	0	0	NA
NRAS	2	0	1	1	NS
PARN	0	0	0	0	NA
PHF6	5	0	5	0	0,0214
PPM1D	0	0	0	0	NA
PRPF8	0	0	0	0	NA
PTEN	0	0	0	0	NA
PTPN11	1	0	0	1	NS
RAD21	1	0	1	0	NS
RIT1	0	0	0	0	NA
RPL11	0	0	0	0	NA
RPL5	0	0	0	0	NA
RTEL1	0	0	0	0	NA
RUNX1	1	0	1	0	ns
SAMD9	0	0	0	0	NA
SAMD9L	0	0	0	0	NA
SBDS	0	0	0	0	NA
SETBP1	0	0	0	0	NA
SF1	0	0	0	0	NA
SF3B1	11	0	5	6	NS
SMC1A	0	0	0	0	NA
SMC3	2	0	2	0	NS
SRP72	0	0	0	0	NA
SRSF2	13	4	8	1	0,0107
STAG2	0	0	0	0	NA
TET2	28	0	28	0	<0,0001
TP53	4	0	2	2	NS
U2AF1	4	0	0	4	NS
U2AF2	0	0	0	0	NA
WT1	0	0	0	0	NA
ZRSR2	3	0	3	0	ns

Fisher's exact test has been realized NS: Not Significative NA: Not Applicable

Supplementary Table 6. NGS at diagnosis in WMC and in NK/T sorted cells.

	VAF White Mononuclear Cells	VAF NK Cells			VAF T Cells	%KIR2D+ NK cells
		Whole	KIR-*	KIR+*		
**MUT22**	Blood 28/02/2017	Blood 31/10/2017				0,60%
TET2:NM_001127208:exon11:c,4669_4672del:p.V1557fs	42	50	NA	NA	-	
SRSF2:NM_001195427:exon1:c.C284T:p.P95L	42	45	NA	NA	-	
TET2:NM_001127208:exon6:c.C3646T:p.R1216X	26	-	NA	NA	-	
TET2:NM_001127208:exon7:c.T3898G:p.F1300V	14	54	NA	NA	-	
ASXL2:NM_018263:exon12:c.1966_1976del:p.T656fs	-	2	NA	NA	-	
NRAS:NM_002524:exon2:c.G35A:p.G12D	-	15	NA	NA	-	
RIT1:NM_006912:exon4:c.G229C:p.A77P	-	7	NA	NA	-	
**MUT33**	Bone Marrow 19/04/2017	Blood 09/01/2018				2,70%
PHF6:NM_001015877:exon2:c.65_66insTA:p.S22fs	91	93	NA	NA	-	
TET2:NM_001127208:exon11:c.4895delA:p.Q1632fs	42	48	NA	NA	-	
TET2:NM_001127208:exon10:c.4400delG:p.R1467fs	41	43	NA	NA	-	
**MUT08**	Blood 18/07/2017	Blood 18/07/2017				10,20%
TET2:NM_001127208:exon9:c.G4108A:p.G1370R	22	49	42	48	-	
TET2:NM_001127208:exon3:c.417delA:p.P139fs	22	33	37	2	-	
SF3B1:NM_012433:exon15:c.A2098G:p.K700E	22	31	33	4	-	
TET2:NM_001127208:intron8:c.4044+2T>A	3	6	8	-	-	
**MUT18**	Bone Marrow 24/03/2010	Blood 06/03/2018				10,70%
TET2:NM_001127208:exon3:c.2050delC:p.Q684fs	36	40	NA	NA	7	
TET2:NM_001127208:exon11:c.5306_5307insTCCGGGCATG:p.A1769fs	34	34	NA	NA	4	
TET2:NM_001127208:exon 3 c.658_663delCACACAinsGAACTTC : p.H220Efs	3	-	NA	NA	-	
TET2:NM_001127208:exon 7 c.3955-2A>G	2	-	NA	NA	-	
KRAS:NM_033360:exon2:c.G34A:p.G12S		12	NA	NA	-	
**MUT24**	Bone Marrow 23/06/16	Bone Marrow 23/06/2016				37,20%
ASXL1:NM_015338:exon12:c.1927dupG:p.G642fs	3	6	6	3	-	
TET2:NM_001127208:exon7:c.T3860C:p.F1287S	2	11	11	7	-	
SF3B1:NM_012433:exon14:c.C1984T:p.H662Y	31	19	20	12	-	
**MUT23**	Bone Marrow 08/12/16	Bone Marrow 08/12/2016				45,90%
TET2:NM_001127208:exon11:c.G5606A:p.G1869E	49	19	9	8	-	
TET2:NM_001127208:exon11:c.T5625A:p.C1875X	49	5	8	13	-	
TET2:NM_001127208:exon11:c.5062_5063del:p.S1688fs	-	-	22	15	-	
TET2:NM_001127208:exon3:c.C1642T:p.Q548X	-	-	6	-	-	
SRSF2:NM_003016:exon1:c.C284T:p.P95L	49	14	29	23	-	
**MUT32**	12/08/2016	Blood 28/12/2017				47,90%
TET2:NM_001127208:exon11:c.A5603G:p.H1868R	46	17	39	3	-	
TET2:NM_001127208:exon11:c.A5770G:p.K1924E	41	20	36	1	-	
SF3B1:NM_012433:exon15:c.A2098G:p.K700E	44	20	34	-	-	
**MUT19**	Blood 08/03/17	Blood 11/01/2018				56,90%
TET2:NM_001127208:exon3:c,C1642T:p.Q548X	32	2	14	-	-	
TET2:NM_001127208:exon11:c.5060_5061del:p.Q1687fs	33	21	31	9	-	
SRSF2:NM_003016:exon1:c.C284A:p.P95H	33	20	29	23	-	
TET2:NM_001127208:exon11:c.5315delT:p.M1772fs	-	4	-	-	-	
**MUT31**	Bone Marrow 14/12/17	Blood 10/03/2016				69,10%
TET2:NM_001127208:exon7:c.G3818C:p.C1273S	34	4	NA	NA	-	
TET2:NM_001127208:exon5:c.T3577A:p.C1193S	38	6	NA	NA	-	
SRSF2:NM_003016:exon1:c.C284A:p.P95H	27	4	NA	NA	-	
CTCF:NM_006565:exon5:c.C1024T:p.R342C	-	-	NA	NA	-	
TET2:NM_001127208:exon3:c.1693_1697del:p.I565fs	-	10	NA	NA	-	
**MUT14**	Blood 22/11/16	Blood 19/12/2017				92,90%
TET2:NM_001127208:exon10:c.C4354T:p.R1452X	16	-	NA	NA	-	
TET2:NM_001127208:exon11:c.5544delA:p.S1848fs	16	-	NA	NA	-	
**PHF6:NM_001015877:exon7:c.T724A:p.C242S**	10	-	NA	NA	-	

*KIR = KIR2D / KIR3DL1 / KIR3DL2
WMC, Whole mononuclear cell; VAF variant allele frequency

Supplementary Table 7. Patients' characteristics.

Variables	All patients (n = 63)	Non *TET2/IDH* mutated patients (n = 30)	*TET2* mutated patients (n = 28)	p=	*IDH* mutated patients (n = 5)
**Age at MDS/CMML diagnostic**	70 [63-75]	71 [62.75-75]	70 [51-86]	ns	73 [59-84.5]
**% women**	38	40	36	ns	40
**Peripheral blood**					
Hb at diagnosis (g/dL)	11.45 [9.35-13.5]	10.15 [9.05-11.88]	13.3 [10.3-14.45]	0.0032	11.2 [9.1-13.4]
Platelets at diagnosis (G/L)	129.5 [89.25-286.8]	124.5 [89.5-369.5]	115 [85-223.5]	ns	155 [108-325.5]
WBC at diagnosis (G/L)	4.8 [3.9-7.4]	4.65 [3.9-7.0]	5 [3.8-7.6]	ns	5.05 [3.6-7.55]
ANC at diagnosis (G/L)	2.7 [1.8-4.2]	2.8 [2.2-4.675]	2.2 [0.6-3.95]	ns	1.8 [1.25-4.75]
Lymphocyte count at diagnosis (G/L)	1.250 [0.9-1.9]	1.2 [0.9-1.7]	1.5 [1.125-2.45]	ns	0.4 [0.2-1.3]
Peripheral blast % at diagnosis (%)	0 [0-0]	0 [0-0]	0 [0-0]	ns	0 [0-0]
**Bone Marrow**					
SLD/MLD*	25	17	6	ns	2
RS-SLD/RS-MLD*	10	5	5	ns	Ø
EB1*	8	4	4	ns	Ø
EB2*	1	1	Ø	ns	Ø
CMML*	17	2	12	0.0018	3
Other MDS/MPN	Ø	Ø	Ø	N.A	Ø
5q-*	2	1	1	ns	Ø
Unknown	Ø	Ø	Ø	N.A	Ø
Medullary blasts (%)	3 [2.0-4.0]	3 [2.0-4.0]	3 [1.0-4.0]	ns	3 [2-3.5]
**IPSS-R**					
Low	52	24	23	ns	5
High	11	6	5	ns	0
**Treatments**	14	5	7	ns	2
Prednisone	4	1	1	ns	2
Lenalidomid	4	3	1	ns	Ø
Rigosertib	1	Ø	1	N.A	Ø
Eltrombopag	1	Ø	1	N.A	Ø
Darbepoietin	4	1	3	ns	Ø

*SLD = MDS with single-lineage dysplasia; MLD = MDS with multilineage dysplasia; RS-SLD = MDS with ring sideroblasts and with single-lineage dysplasia; RS-MLD = MDS with ring sideroblasts and with multilineage dysplasia); MPN = Myeloproliferative Neoplasm; del5q = MDS with isolated del(5q); EB1 = MDS with excess blasts (5 to 9 percent blasts in the bone marrow or 2 to 4 percent blasts in the blood); EB2 = MDS with excess blasts (10 to 19 percent blasts in the bone marrow or 5 to 19 percent blasts in the blood); CMML = Chronic Myelomonocytic Leukemia.

Supplementary Table 8. Patients of the Clinical Trial NCT02985190

Code	**Patient number (in Mekinian et al. Leukemia 2022)**	**Age**	**Sex**	**WHO 2016 classification***	**Karyotype**	**Mutations (Targeted NGS)**	**IPSS-R**
MUT34	#24	73	F	MDS-U	45,X,-Y[19]/46,XY[5]	*TET2, ASXL1, CBL, JAK2, PHF6*	2.0
MUT35	#14	72	M	MDS-EB2	46,xy[20]	*TET2, DNMT3A, TP53*	9.0
MUT36	#18	86	F	CMML-1	46,XY,del(20)(q11q13)[1]/46,XY[1]	*TET2, NRAS, KRAS, CBL*	2.0
WT31	#21	77	F	CMML-2	46,XXdel(5)(q13q33)	*SF3B1, NRAS, RUNX1, SETBP1*	4.5
WT32	#29	76	M	MDS-MLD	46,XY,del(13)(q13q21)[3]/46,XY[17]	*UBA1*	2.0
WT33	#26	68	M	MDS-MLD	46,X,inv(Y)(p11q12)c[20]	*UBA1*	4.5
WT34	#16	69	M	MDS-MLD	46,XX,del(3)(p21)[3]/46,XX[20]	*UBA1*	1.0
WT35	#22	82	M	MDS-MLD	92,XXXX[3] / 46,XX[17]	*UBA1*	3
WT36	#11	82	M	MDS-MLD	45,X, -Y[20]	*DNMT3A*	2.5
WT37	#16	69	M	MDS-MLD	46,XX,del(3)(p21)[3]/46,XX[20]	*UBA1*	1.0

*SLD = MDS with single-lineage dysplasia; MLD = MDS with multilineage dysplasia; RS-SLD = MDS with ring sideroblasts and with single-lineage dysplasia; RS-MLD = MDS with ring sideroblasts and with multilineage dysplasia; del5q = MDS with isolated del(5q); EB1 = MDS with excess blasts (5 to 9 percent blasts in the bone marrow or 2 to 4 percent blasts in the blood); EB2 = MDS with excess blasts (10 to 19 percent blasts in the bone marrow or 5 to 19 percent blasts in the blood). CMML-1 = Chronic Myelomonocytic Leukemia (5 to 9 percent blasts in the bone marrow or 2 to 4 percent blasts in the blood). CMML-2 = Chronic Myelomonocytic Leukemia (10 to 19 percent blasts in the bone marrow or 5 to 19 percent blasts in the blood).

Supplementary Table 9. List of Antibodies used for Flow Cytometry.

Target	Conjugaison	Clone	Supplier	Reference
CD3	FITC	OKT3	Biolegend	317306
CD3	V450	UCHT1	BD	560365
CD3	BB700	SK7	BD	566575
CD4	FITC	OKT4	Biolegend	317408
CD4	APC-H7	RPA-T4	BD	560158
CD5	FITC	UCHT2	Biolegend	300606
CD7	PE-CF594	M-T701	BD	562541
CD7	APC	CD7-6B7	Miltenyi	130-105-841
CD8	BV785	SK1	Biolegend	344740
CD14	FITC	M5E2	BD	555397
CD16	BUV395	3G8	BD	563785
CD16	APC-H7	3G8	BD	560715
CD16	BV711	3G8	BD	563127
CD19	FITC	HIB19	Biolegend	302206
CD33	PE	WM53	BD	555450
CD56	AF700	B159	BD	557919
CD56	PE-Cy7	B159	BD	557747
CD56	BV421	HCD56	Biolegend	318328
CD57	BV605	QA17A04	Biolegend	393304
CD69	BUV737	FN50	BD	564439
CD85j	PE-Cy5	GHI/75	BD	551054
CD96	APC	NK92.39	Biolegend	338410
CD107a	APC-H7	H4A3	BD	561343
DNAM-1	PE-Vio770	REA1040	Miltenyi	130-099-966
KLRG1	APC-Vio770	REA260	Miltenyi	130-103-642
NKG2A	BV786	131411	BD	747917
NKG2D	BV650	1D11	BD	563408
NKp30	BV421	p30-15	BD	563385
NKp46	BV786	9-E2	BD	563329
CD127	PE-Cy7	A019D5	Biolegend	351320
IFN-γ	AF488	B27	BD	557718
CD158a (KIR2DL1)	APC	REA284	Miltenyi	130-103-935
CD158b (KIR2DL2/DL3)	BB700	CH-L	BD	746236
CD158d (KIR2DL4)	PE	REA768	Miltenyi	130-112-465
CD158e/k (KIR3DL1/DL2)	PE	5.133	Miltenyi	130-095-205
CD158e/k (KIR3DL1/DL2)	APC-Vio770	REA970	Miltenyi	130-116-181
CD158e1/e2 (KIR3DL1/DS1	PerCP-Vio70	REA168	Miltenyi	130-104-837
CD158f (KIR2DL5)	PE-Vio770	REA955	Miltenyi	130-115-841
CD158i (KIR2DS4)	VioBlue	REA860	Miltenyi	130-114-622
KIR2D (all KIR2D)	PE	NKVFS1	Miltenyi	130-092-688
KIR2D (all KIR2D)	APC	NKVFS1	Miltenyi	130-092-687

Supplementary Table 10. List of primers used for the ChIP-qPCR assay

ID	Sequence
CRE Forward	GGGGAGGGCAGTTTCCGTTC
CRE Reverse	GGTCATGGCGTCTCCTCTGC
KIR2DL1 Forward	GGCCTCATGCAAGGTAGAAA
KIR2DL1 reverse	CCCTCCCTCTATTGCTTTCC
KIR2DL2/DL3 Forward	CTCTTGAGCGAGCACCCAC
KIR2DL1prom2 Reverse	CACCAACACACACCATGCTG

Supplemental Table 11. Specificities and primers used in the Multiplex RT-PCR analysis.

Target Gene	Reference	Target Gene	Reference
TET2	Hs00325999_m1	*IKZF3*	Hs05037772_s1
IDH1	Hs00271858_m1	*ZBTB16*	Hs00232313_m1
IDH2	Hs00158033_m1	*IRF2*	Hs01082884_m1
CXCL10	Hs00171042_m1	*JUN*	Hs01103582_s1
RUNX1	Hs01021970_m1	*FOS*	Hs99999140_m1
MYC	Hs00153408_m1	*DUSP5*	Hs00244839_m1
DNMT3A	Hs01027166_m1	*EOMES*	Hs00172872_m1
TET1	Hs00286756_m1	*NFIL3*	Hs00705412_s1
TET3	Hs00379125_m1	*PRDM1*	Hs00153357_m1
PRF1	Hs00169473_m1	*STAT3*	Hs00374280_m1
GZMA	Hs00989184_m1	*STAT4*	Hs01028017_m1
GZMB	Hs00188051_m1	*STAT5B*	Hs00560026_m1
GZMK	Hs00157878_m1	*STAT6*	Hs00598625_m1
GZMH	Hs00277212_m1	*ID2*	Hs04187239_m1
INFG	Hs00989291_m1	*ID3*	Hs00171409_m1
TNF	Hs00174128_m1	*BCL6*	Hs00153368_m1
BCL2	Hs00608023_m1	*ZEB1*	Hs01566410_m1
KIR2DL1	Hs04961778_gH	*ZEB2*	Hs00207691_m1
KIR2DL2	Hs04961776_uH	*BCL6*	Hs00158218_m1
KIR2DL3	Hs04961777_gH	*LEF1*	Hs01547250_m1
KIR2DL4	Hs00427106_m1	*TCF7*	Hs00175273_m1
KIR3DL1	Hs01592437_m1	*TNFRSF18*	Hs00188346_m1
KIR3DL2	Hs00601497_gH	*EZH2*	Hs00544830_m1
KIR3DL3	Hs00601067_m1	*MYB*	Hs00920556_m1
CXCR4	Hs00976734_m1	*GAPDH*	Hs99999905_m1
ACKR3	Hs00664172_s1	*ACTB*	Hs99999903_m1
ITGA4	Hs00168433_m1	*RPL27*	Hs03044961_g1
KLRC1	Hs00970273_g1	*NCAM1*	Hs00941830_m1
CD96	Hs00976975_m1	*FCGR3B*	Hs04334165_m1
CD69	Hs00934033_m1	*CD3E*	Hs01062241_m1
KLRG1	Hs00195153_m1	*CD4*	Hs01058407_m1
CXCR3	Hs01847760_s1	*CD8B*	Hs00174762_m1
CCR2	Hs00704702_s1	*CD33*	Hs00233544_m1
NCR2	Hs00183113_m1	*CD5*	Hs00204397_m1
NCR3	Hs00394809_m1	*CD7*	Hs00196191_m1
NCR1	Hs00183118_m1	*SPI1*	Hs02786711_m1
TIGIT	Hs00545087_m1	*NT5E*	Hs00159686_m1
CD226	Hs00170832_m1	*ENTPD1*	Hs00969556_m1
IL2RA	Hs00907777_m1	*CTLA4*	Hs00175480_m1
IL7R	Hs00902334_m1	*HAVCR2*	Hs00958618_m1
IL15RA	Hs00542602_g1	*PDCD1*	Hs01550088_m1
AHR	Hs00169233_m1	*LAG3*	Hs00958444_g1
CYP1A1	Hs01054796_g1	*CD244*	Hs00175569_m1
NQO1	Hs01045993_g1	*FN1*	Hs01549976_m1
TBX21	Hs00203436_m1	*CD44*	Hs01075864_m1
FOXO1	Hs00231106_m1	*ITGA4*	Hs00168433_m1
FOXO3	Hs00818121_m1	*ITGB1*	Hs01127536_m1
ELF4	Hs01086126_m1	*SELL*	Hs00174151_m1
