# Supplementary material for: Myelodysplastic Syndrome associated TET2 mutations affect NK cell function and genome methylation
Source: Nat Commun. 2023 Feb 3;14:588. doi: 10.1038/s41467-023-36193-w (PMC9898569; doi:10.1038/s41467-023-36193-w)
Supplement: Supplementary file 2 — Description of Additional Supplementary Files [file 41467_2023_36193_MOESM2_ESM.pdf]

Myelodysplastic Syndrome associated TET2 mutations affect NK cell function and genome methylation

Description of Additional Supplementary Files

File Name: Supplementary Data1.
Description: MDS/CMML patients' individual clinical information.

File Name: Supplementary Data 2.
Description: List of 15,827 Differentially Methylated Sites.

File Name: Supplementary Data 3.
Description: List of 117 Differentially Methylated Genes.
