# Supplementary material for: Myelodysplastic Syndrome associated TET2 mutations affect NK cell function and genome methylation
Source: Nat Commun. 2023 Feb 3;14:588. doi: 10.1038/s41467-023-36193-w (PMC9898569; doi:10.1038/s41467-023-36193-w)
Supplement: Supplementary file 6 — Reporting Summary [file 41467_2023_36193_MOESM6_ESM.pdf]

nature portfolio

Corresponding author(s): DULPHY Nicolas

Last updated by author(s): Dec 30, 2022

Reporting Summary

Nature Portfolio wishes to improve the reproducibility of the work that we publish. This form provides structure for consistency and transparency in reporting. For further information on Nature Portfolio policies, see our Editorial Policies and the Editorial Policy Checklist.

Statistics

For all statistical analyses, confirm that the following items are present in the figure legend, table legend, main text, or Methods section.

n/a	Confirmed	
☐	☒	The exact sample size (*n*) for each experimental group/condition, given as a discrete number and unit of measurement
☐	☒	A statement on whether measurements were taken from distinct samples or whether the same sample was measured repeatedly
☐	☒	The statistical test(s) used AND whether they are one- or two-sided<br>*Only common tests should be described solely by name; describe more complex techniques in the Methods section.*
☐	☒	A description of all covariates tested
☒	☐	A description of any assumptions or corrections, such as tests of normality and adjustment for multiple comparisons
☐	☒	A full description of the statistical parameters including central tendency (e.g. means) or other basic estimates (e.g. regression coefficient) AND variation (e.g. standard deviation) or associated estimates of uncertainty (e.g. confidence intervals)
☐	☒	For null hypothesis testing, the test statistic (e.g. *F*, *t*, *r*) with confidence intervals, effect sizes, degrees of freedom and *P* value noted<br>*Give P values as exact values whenever suitable.*
☒	☐	For Bayesian analysis, information on the choice of priors and Markov chain Monte Carlo settings
☒	☐	For hierarchical and complex designs, identification of the appropriate level for tests and full reporting of outcomes
☐	☒	Estimates of effect sizes (e.g. Cohen's *d*, Pearson's *r*), indicating how they were calculated

Our web collection on statistics for biologists contains articles on many of the points above.

Software and code

Policy information about availability of computer code

Data collection	FACS Diva software version 7 (BD), SpectroFlo v3.0.3 (Cytek)
Data analysis	FlowJo v10.7 (BD), GraphPad Prism v8.0e (GraphPad Software Inc.), SpectroFlo v3.0.3 (Cytek), cBioPortal Cancer Genomics Portal OncoPrinter software (https://www.cbioportal.org/), St Jude protein paint software (https://proteinpaint.stjude.org/), Bismark v0.20.0 (https://www.bioinformatics.babraham.ac.uk/projects/bismark/)

For manuscripts utilizing custom algorithms or software that are central to the research but not yet described in published literature, software must be made available to editors and reviewers. We strongly encourage code deposition in a community repository (e.g. GitHub). See the Nature Portfolio guidelines for submitting code & software for further information.

Data

Policy information about availability of data

All manuscripts must include a data availability statement. This statement should provide the following information, where applicable:

- Accession codes, unique identifiers, or web links for publicly available datasets
- A description of any restrictions on data availability
- For clinical datasets or third party data, please ensure that the statement adheres to our policy

All sequencing data were analysed using the human reference genome hg38/GRCh38.
Reduced representation bisulfite sequencing (RRBS) data that support the findings of this study have been deposited in the Gene Expression Omnibus data

repository with the accession number GSE183020 (http://www.ncbi.nlm.nih.gov/geo/query/acc.cgi?acc=GSE183020). The in vitro data generated in this study are available in the Supplementary information. Source data are provided with this paper as a Source Data file.

Human research participants

Policy information about studies involving human research participants and Sex and Gender in Research.

Reporting on sex and gender	Sex (biological attribute) of individuals recruited in the study was considered as a covariate-relevant population characteristic of the participants. Sex of participants has been determined on self-report. Overall 24 (38%) women and 39 (62%) men have been recruited with a similar distribution across patients' groups.
Population characteristics	Cohort was composed of sternal bone marrow aspiration (BM) and peripheral blood mononuclear cell (PBMCs) samples that were collected from patients with MDS/CMML at the Hematological Departments on the Saint Louis Hospital (Paris, France). Individual patients clinical characteristics and cohort characteristics are detailed in Supplemental Table 1 and 10 respectively. Population characteristics relevant for the study were age, sex (biological attribute), diagnosis of MDS/CMML, somatic mutation pattern of the disease, IPSS-R scoring of the disease, and treatments.
Recruitment	Healthy individuals were randomly recruited based on the sample availability in the Saint-Louis hospital blood bank facility. Patients were recruited based on the diagnosis of MDS/CMML according to the World Health Organization 2016 criteria, and classified according to the Revised International Prognostic Scoring System (IPSS-R). Recruited patients gave their written informed consent for participating to the study.
Ethics oversight	The study was approved by the Ethical Board Ile-de-France X. The study was conducted in accordance with Helsinki's declaration. All patients recruited for the biological analysis gave their written informed consent for this procedure.

Note that full information on the approval of the study protocol must also be provided in the manuscript.

Field-specific reporting

Please select the one below that is the best fit for your research. If you are not sure, read the appropriate sections before making your selection.

☒ Life sciences ☐ Behavioural & social sciences ☐ Ecological, evolutionary & environmental sciences

For a reference copy of the document with all sections, see nature.com/documents/nr-reporting-summary-flat.pdf

Life sciences study design

All studies must disclose on these points even when the disclosure is negative.

Sample size	63 patients were collected and our cohort was separated in two group according to the presence of TET2/IDH mutation : 33 were TET2/IDH mutated and 30 were TET2/IDH wt. Median of age and sex-ratio was comparable between the two group.<br>No statistical methods were used to determine sample sizes. For both ex vivo and in vitro experiments, sample sizes were determined based on the number of samples available combined with the amount of samples necessary to determine statistical significance.
Data exclusions	No data were excluded in this study.
Replication	All attempts at replication were successful.<br>In vitro assays were repeated in at least three or more independent experiments. Because of the availability of the patients' samples and the expense, RRBS experiment has been carried out once.
Randomization	Samples were allocated into experimental groups based on their diagnosis (i.e. healthy individuals of MDS/CMML patients) and, for MDS/CMML patients, on the TET2/IDH mutation status. Moreover, patient's mutation status was known after the ex vivo phenotyping experiments were performed. Other covariates, such as sex or age, were not used to alocate MDS/CMML patients in experimental groups, as they are not relevant for the history of the disease.
Blinding	Ex vivo phenotyping experiments were performed at diagnosis, before any indication of the somatic mutation status. Therefore, the analysis of the ex vivo experiments was blinded. By contrast, blinding for in vitro experiments was impractical.

Reporting for specific materials, systems and methods

We require information from authors about some types of materials, experimental systems and methods used in many studies. Here, indicate whether each material, system or method listed is relevant to your study. If you are not sure if a list item applies to your research, read the appropriate section before selecting a response.

Materials & experimental systems

n/a	Involved in the study	
☐	☒	Antibodies
☐	☒	Eukaryotic cell lines
☒	☐	Palaeontology and archaeology
☒	☐	Animals and other organisms
☐	☒	Clinical data
☒	☐	Dual use research of concern

Methods

n/a	Involved in the study	
☒	☐	ChIP-seq
☐	☒	Flow cytometry
☒	☐	MRI-based neuroimaging

Antibodies

Antibodies used

Antibodies used in flow-cytometry experiments:
Antibodies as recommended by the supplier according to the technical data sheet provided with the reagent.

Target Conjugaison Clone Supplier Reference
CD3 FITC OKT3 Biolegend 317306
CD3 V450 UCHT1 BD 560365
CD3 BB700 SK7 BD 566575
CD4 FITC OKT4 Biolegend 317408
CD4 APC-H7 RPA-T4 BD 560158
CD5 FITC UCHT2 Biolegend 300606
CD7 PE-CF594 M-T701 BD 562541
CD7 APC CD7-6B7 Miltenyi 130-105-841
CD8 BV785 SK1 Biolegend 344740
CD14 FITC M5E2 BD 555397
CD16 BUV395 3G8 BD 563785
CD16 APC-H7 3G8 BD 560715
CD16 BV711 3G8 BD 563127
CD19 FITC HIB19 Biolegend 302206
CD33 PE WM53 BD 555450
CD56 AF700 B159 BD 557919
CD56 PE-Cy7 B159 BD 557747
CD56 BV421 HCD56 Biolegend 318328
CD57 BV605 QA17A04 Biolegend 393304
CD69 BUV737 FN50 BD 564439
CD85j PE-Cy5 GHI/75 BD 551054
CD96 APC NK92.39 Biolegend 338410
CD107a APC-H7 H4A3 BD 561343
DNAM-1 PE-Vio770 REA1040 Miltenyi 130-099-966
KLRG1 APC-Vio770 REA260 Miltenyi 130-103-642
NKG2A BV786 131411 BD 747917
NKG2D BV650 1D11 BD 563408
NKp30 BV421 p30-15 BD 563385
NKp46 BV786 9-E2 BD 563329
CD127 PE-Cy7 A019D5 Biolegend 351320
IFN-γ AF488 B27 BD 557718
CD158a (KIR2DL1) APC REA284 Miltenyi 130-103-935
CD158b (KIR2DL2/DL3) BB700 CH-L BD 746236
CD158d (KIR2DL4) PE REA768 Miltenyi 130-112-465
CD158e/k (KIR3DL1/DL2) PE 5.133 Miltenyi 130-095-205
CD158e/k (KIR3DL1/DL2) APC-Vio770 REA970 Miltenyi 130-116-181
CD158e1/e2 (KIR3DL1/DS1) PerCP-Vio700 REA168 Miltenyi 130-104-837
CD158f (KIR2DL5) PE-Vio770 REA955 Miltenyi 130-115-841
CD158i (KIR2DS4) VioBlue REA860 Miltenyi 130-114-622
KIR2D (all KIR2D) PE NKVFS1 Miltenyi 130-092-688
KIR2D (all KIR2D) APC NKVFS1 Miltenyi 130-092-687

Antibodies used for Chromatin Immuno-Precipitation (ChIP)-qPCR assay:
Antibodies as recommended by the supplier according to the technical data sheet provided with the reagent.

Target Clone Supplier Reference
TET2 Rabbit-Polyclonal Diagenode C15410255 (5µg used per experiment)
H3 Rabbit-Polyclonal Abcam AB1791 (2µg used per experiment)
H3K18 Rabbit-Polyclonal Abcam AB1191 (2µg used per experiment)

Validation

All monoclonal antibodies are validated for use in flow cytometry.
All rabbit polyclonal antibodies are validated for ChIP assays.
Data are available on the manufacturer's websites. The antibodies have been validated by the manufacturers. No additional validation was carried out.

Eukaryotic cell lines

Policy information about cell lines and Sex and Gender in Research

Cell line source(s)	K562 (ATCC)<br>HEK293T (ATCC)<br>JURKAT (ATCC)
Authentication	Cells were authenticated by the supplier. No other authentications were performed.
Mycoplasma contamination	All cell lines were tested negative for mycoplasma.
Commonly misidentified lines (See ICLAC register)	No misidentified cells lines were used in the study.

Clinical data

Policy information about clinical studies

All manuscripts should comply with the ICMJE guidelines for publication of clinical research and a completed CONSORT checklist must be included with all submissions.

Clinical trial registration	#NCT02985190
Study protocol	Azacitidine 75mg/m²/j subcutaneously daily for 7 days every 4 weeks for a minimum of 6 cycles (unless overt disease progression, especially to Acute Myeloid Leukemia (AML) occure before 6 cycles).<br>Azacitidine will be continued after 6 cycles:<br>- in patients with hematological response of myelodysplastic syndrome to azacitidine according to IWG2006 criteria by 6 cycles (Complete Response (CR), Partial Response (PR), marrow Complete Response (CRm), stable disease with Hematological Improvment (HI)), for another 6 cycles<br>- in patients with complete or partial response of Systemic Auto-Immune Disorders (SAID) after 6 cycles of Azacitidine, even if Myelodysplastic Syndrome remains only stable per IWG2006 criteria.<br><br>Full trial protocol can be accessed on the ClinicalTrials.gov website (https://clinicaltrials.gov/ct2/show/NCT02985190).
Data collection	Data were collected by the French-speaking Group for Myelodysplasia (GFM). Anonymous data collection was declared to the relevant French authorities.<br>Thirty patients were included between July 2017 and June 2020 in 18 centers and data were collected until January 2022. Data were collected on the register website of the French-speaking Group for Myelodysplasia (http://www.gfmgroup.org/registre.php) following a secured authentication protocol.
Outcomes	Primary outcome : Overall response rate of Myelodysplastic syndrome and systemic autoimmune and inflammatory diseases (SAID).<br>Secondary outcome : Number of participants with treament-related adverse events as assessed by CTCAE v4.0.<br>Of note, it needs to be noticed that the manuscript is not dedicated to present any clinical results of the trial #NCT02985190. Results of the trial have been published in "Mekinian, A., Zhao, L.P., Chevret, S. et al. A Phase II prospective trial of azacitidine in steroid-dependent or refractory systemic autoimmune/inflammatory disorders and VEXAS syndrome associated with MDS and CMML. Leukemia (2022). DOI: 10.1038/s41375-022-01698-8".

Flow Cytometry

Plots

Confirm that:

☒ The axis labels state the marker and fluorochrome used (e.g. CD4-FITC).

☒ The axis scales are clearly visible. Include numbers along axes only for bottom left plot of group (a 'group' is an analysis of identical markers).

☒ All plots are contour plots with outliers or pseudocolor plots.

☒ A numerical value for number of cells or percentage (with statistics) is provided.

Methodology

Sample preparation	Mononuclear cells were purified from bone marrow and blood samples by using gradient density methods as previously described and stored in liquid nitrogen. Cells were thawed and each sample was used in various experiments according to its availability.<br>Frozen sternal BM aspiration and PBMCs were thawed at 37°C and then resuspended in RPMI 1640 (Life Technologies) supplemented with 10% FBS (Life Technologies), Penicillin-Streptomycin (Dutscher), L-glutamine (Life Technologies), Sodium Pyruvate (EuroBio), HEPES (EuroBio). Cells were treated for 15 min with 1000 U/ml of DNAse I. PBMCs and BM cells were first labeled in PBS 30min at 4°C with the fixable viability dye eFluor 506 (eBioscience), and then were stained in the same conditions in presence of brilliant stain buffer (Becton Dickinson Biosciences) with specific antibodies of surface markers. Foxp3 Transcription factor buffer set (eBioscence 00-5523-00) was used for intracellular staining of granzyme B, perforin and IFN-γ. Samples were first stained for surface markers (CD3, CD56, CD16, KIR2D, CD158e1_e2 = KIR3DL1/DS1) as described

above, then fixed and permeabilized with fixation/permeabilization solution 1X, labelled with perforin and granzyme antibodies, washed with permeabilization buffer 1X and were fixed in PBS-2% PFA.

Instrument	BD LSRFortessa™ Flow Cytometer, BD FACSAria™ III Cell Sorter, BD FACSCanto™ II Cell Analyzer, Cytek Aurora Spectral Flow Cytometer.
Software	The FACS diva software v7 (BD) and the Cytek SpectroFlo v3.0.3 software were used to collect data. The FlowJo v10.7 software and the Cytek SpectroFlo v3.0.3 software were used for data analysis.
Cell population abundance	NK cells represented approximatively 1-5% of total cells. T cells represented approximatively 10-20% of total cells.
Gating strategy	All gating strategy starded by :<br>1. Gate on FSC-A vs SSC-A was set to include leukocytes but excluding debris<br>2. Gate on FSC-A vs FSC-H was set to exclude doublets.<br>3. Gate on SSC-A vs SSC-H was set to exclude doublets.<br>4.Gate on SSC-A vs E506 Viability Dye was set to exclude dead cells (E506+)<br><br>Gating for NK cells Phenotyping, Cell sorting, Multiplex RT-QPCR.<br>5. Gate on Lineage vs CD7 to include all innate lymphoid cells (CD7+lin-)<br>6.Gate on CD56 vs CD16 to include All NK cells (CD56+ CD16+/-)<br><br>Gating for T cells NGS and RRBS<br>5. Gate on Lineage* vs SCC to exclude all non lymphoid cells (lin*-)<br>6.Gate on CD3 vs CD7 to include all T cells (CD3+ CD7+)

☒ Tick this box to confirm that a figure exemplifying the gating strategy is provided in the Supplementary Information.
